# A Sodium-Translocating Module Linking Succinate Production to Formation of Membrane Potential in Prevotella bryantii

**DOI:** 10.1128/AEM.01211-21

**Published:** 2021-10-14

**Authors:** Lena Schleicher, Andrej Trautmann, Dennis P. Stegmann, Günter Fritz, Jochem Gätgens, Michael Bott, Sascha Hein, Jörg Simon, Jana Seifert, Julia Steuber

**Affiliations:** a Hohenheim Center for Livestock Microbiome Research, University of Hohenheim, Stuttgart, Germany; b Institute of Biology, University of Hohenheim, Stuttgart, Germany; c Institute of Animal Science, University of Hohenheim, Stuttgart, Germany; d Microbial Energy Conversion and Biotechnology, Department of Biology, Technical University of Darmstadt, Darmstadt, Germany; e Institute of Bio- and Geosciences, IBG-1: Biotechnology, Forschungszentrum Jülich, Jülich, Germany; Wageningen University

**Keywords:** Na^+^-translocating NADH:quinone oxidoreductase, fumarate reductase, supercomplex, *Prevotella bryantii*, rumen, anaerobic respiration

## Abstract

Ruminants such as cattle and sheep depend on the breakdown of carbohydrates from plant-based feedstuff, which is accomplished by the microbial community in the rumen. Roughly 40% of the members of the rumen microbiota belong to the family *Prevotellaceae*, which ferments sugars to organic acids such as acetate, propionate, and succinate. These substrates are important nutrients for the ruminant. In a metaproteome analysis of the rumen of cattle, proteins that are homologous to the Na^+^-translocating NADH:quinone oxidoreductase (NQR) and the quinone:fumarate reductase (QFR) were identified in different *Prevotella* species. Here, we show that fumarate reduction to succinate in anaerobically growing Prevotella bryantii is coupled to chemiosmotic energy conservation by a supercomplex composed of NQR and QFR. This sodium-translocating NADH:fumarate oxidoreductase (SNFR) supercomplex was enriched by blue native PAGE (BN-PAGE) and characterized by in-gel enzyme activity staining and mass spectrometry. High NADH oxidation (850 nmol min^−1 ^mg^−1^), quinone reduction (490 nmol min^−1 ^mg^−1^), and fumarate reduction (1,200 nmol min^−1 ^mg^−1^) activities, together with high expression levels, demonstrate that SNFR represents a charge-separating unit in *P. bryantii*. Absorption spectroscopy of SNFR exposed to different substrates revealed intramolecular electron transfer from the flavin adenine dinucleotide (FAD) cofactor in NQR to heme *b* cofactors in QFR. SNFR catalyzed the stoichiometric conversion of NADH and fumarate to NAD^+^ and succinate. We propose that the regeneration of NAD^+^ in *P. bryantii* is intimately linked to the buildup of an electrochemical gradient which powers ATP synthesis by electron transport phosphorylation.

**IMPORTANCE** Feeding strategies for ruminants are designed to optimize nutrient efficiency for animals and to prevent energy losses like enhanced methane production. Key to this are the fermentative reactions of the rumen microbiota, dominated by *Prevotella* spp. We show that succinate formation by *P. bryantii* is coupled to NADH oxidation and sodium gradient formation by a newly described supercomplex consisting of Na^+^-translocating NADH:quinone oxidoreductase (NQR) and fumarate reductase (QFR), representing the sodium-translocating NADH:fumarate oxidoreductase (SNFR) supercomplex. SNFR is the major charge-separating module, generating an electrochemical sodium gradient in *P. bryantii*. Our findings offer clues to the observation that use of fumarate as feed additive does not significantly increase succinate production, or decrease methanogenesis, by the microbial community in the rumen.

## INTRODUCTION

Human nutrition heavily depends on ruminants such as cattle and sheep, which require nutrients provided by their rumen microbiota in the course of anoxic degradation of feed (1). In the rumen, a consortium of anaerobic microorganisms provides enzymes and metabolic pathways for the degradation of plant-based feed. The rumen microbiome is highly similar across the globe (2) and offers energy-rich substrates such as acetate, succinate, and propionate to the ruminant. These carboxylic acids are typical electron sinks released by fermenting bacteria to maintain their internal redox balance. The complete breakdown of complex organic matter such as cellulose under anoxic conditions in the rumen requires primary and secondary fermenting bacteria, as well as methanogenic archaea (3). In fact, the formation of methane is the main electron sink in the ruminal degradation of carbohydrates. Its release to the atmosphere diminishes the energy available to the ruminant (4) and has a negative impact on global climate (5). Members of the family *Prevotellaceae* represent the most abundant organisms in the rumen (1, 4). The metabolism of *Prevotella* spp. is expected to have a strong impact on overall carbon flux in the rumen ecosystem and on the nutrition of the host, the ruminant.

In the past, *Prevotella* spp. were thought to depend exclusively on ATP generation by substrate-level phosphorylation (6). Proteome analysis of the rumen microbiota from dairy cows (7) and studies with Prevotella bryantii (7), Prevotella copri (8) and Bacteroides fragilis (9) suggested an important catabolic role of two enzymes, the Na^+^-translocating NADH:quinone oxidoreductase (NQR) and the fumarate:menaquinol oxidoreductase (QFR) producing succinate, which is excreted from the cells. NQR is a respiratory enzyme composed of six subunits (NqrABCDEF) embedded in the inner membrane of several Gram-negative bacteria, including human pathogens such as Vibrio cholerae (10). NQR from *P. bryantii* exhibits high sequence similarity to the V. cholerae NQR (7), including the conserved domains for the binding of six cofactors: one flavin adenine dinucleotide (FAD), two iron-sulfur centers, one riboflavin, and two covalently bound flavin mononucleotides (FMNs) (10, 11). The NqrF subunit harbors the FAD and is the entry point for the electrons into the protein complex, oxidizing NADH to NAD^+^. NqrA was shown to interact with ubiquinone-8 (12) and is therefore most likely responsible for the reduction of quinone to quinol as a final step of the electron transfer within NQR. Exergonic NADH:quinone oxidoreduction drives endergonic transport of sodium ions from the cytoplasm to the periplasm through NqrB (11), resulting in an electrochemical Na^+^ gradient. The *P. bryantii* QFR is highly similar to the type B class of QFRs (7, 13), exemplified by the enzyme from Wolinella succinogenes. Sequence comparisons suggest that the subunit FrdA contains one covalently bound flavin close to the fumarate binding site and that FrdB has three iron-sulfur clusters. FrdA and FrdB presumably interact with membrane-bound FrdC, which contains two hemes *b* and offers a binding site for menaquinol (14). QFR operates in fumarate respiration with NADH as an electron donor under generation of a transmembrane voltage (15). QFR also participates in the reductive branch of mixed-acid fermentation leading to succinate (16).

Here, we demonstrate the existence of a sodium-translocating supercomplex consisting of NQR and QFR in the membrane of *P. bryantii* which we term SNFR (sodium-translocating NADH:fumarate oxidoreductase). Respiratory supercomplexes in prokaryotes (17, 18) and in the inner membrane of mitochondria (19) have been known for many years. In *P. bryantii*, the SNFR couples NADH oxidation with reduction of fumarate to succinate to generate a membrane potential. The implications of our findings for the metabolism of *P. bryantii* and for carbon flux in the rumen ecosystem are discussed.

## RESULTS

### Reduction of fumarate during glucose fermentation by *P. bryantii*.

The presence of gene clusters coding for membrane-bound redox enzymes (see Fig. S1 in the supplemental material) raised the question of the composition and metabolic function of an electron transport chain in *P. bryantii*. In proteomic studies with *P. bryantii* (7), NQR and QFR subunits were identified which exhibit sequence similarity to V. cholerae NQR and *W. succinogenes* QFR, respectively (Fig. S2 and S3). *P. bryantii* lacks genes coding for hydrogenases and formate dehydrogenases. Further electron acceptors to be considered are nitrate, sulfate, and elemental sulfur. The genome of *P. bryantii* (Joint Genome Institute Integrated Microbial Genomes [JGI IMG]) lacks genes coding for enzymes involved in dissimilatory nitrate reduction (nitrate reductase, nitrite reductase, N_2_O reductase, and NO reductase), sulfate reduction (ATP sulfurylase, phosphoadenosine phosphosulfate reductase, and sulfite reductase), or sulfur reduction (polysulfide reductase). Genes conferring the ability to utilize acceptors such as manganese, iron, and cobalt are also absent in *P. bryantii*. These findings are in accordance with our previous proteomic study (7). Thus, only fumarate is predicted based on annotation to serve as a terminal electron acceptor in *P. bryantii*, which is reduced by quinol:fumarate oxidoreductase (QFR).

**FIG 1:**
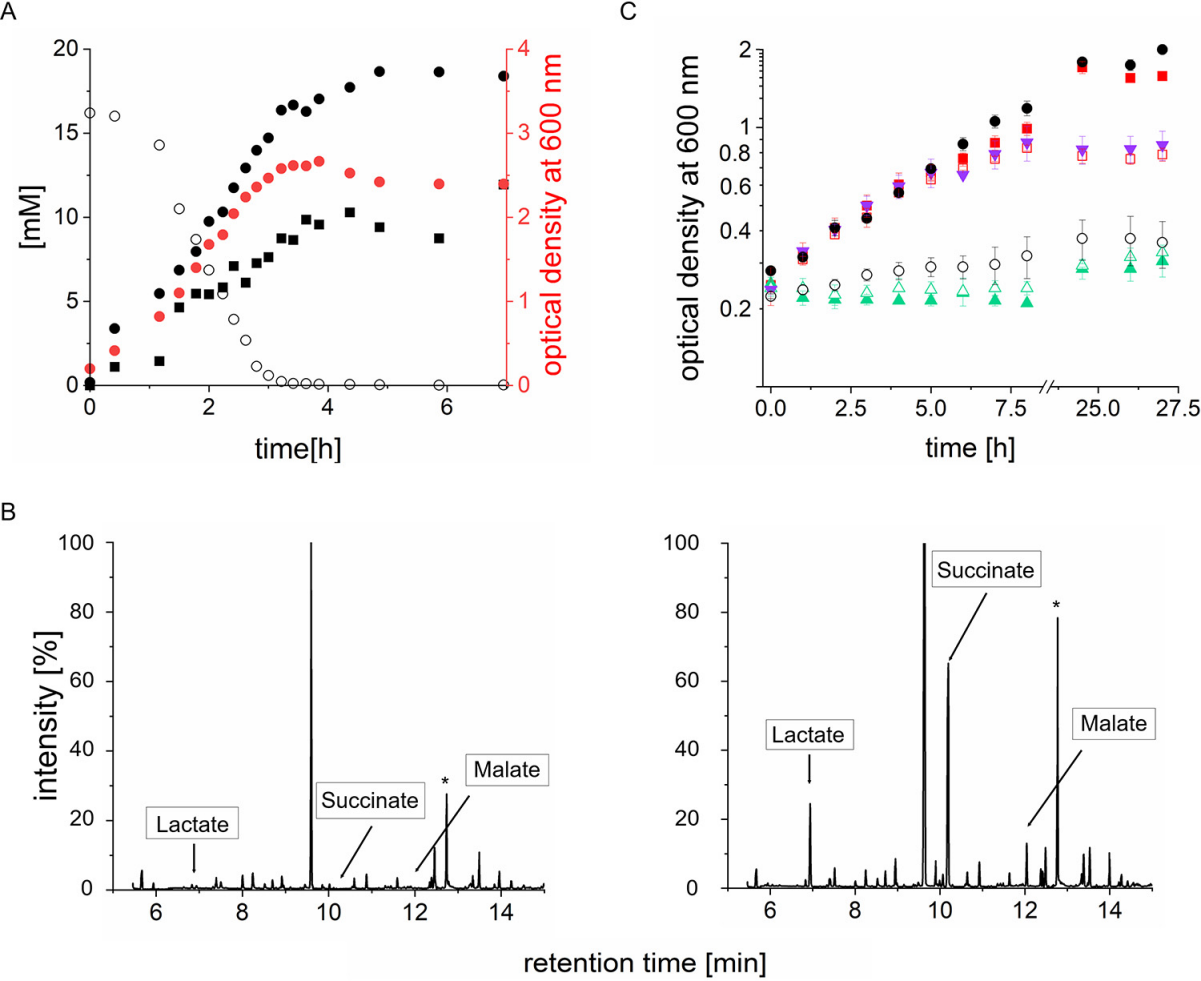
Growth of *P. bryantii*. (A) Concentrations of glucose (open circles), succinate (black circles), and acetate (black squares) and optical density at 600 nm (red circles) were monitored during growth in tryptone- and glucose-containing medium (2 liters). (B) GC-TOF mass spectrometry of metabolites in *P. bryantii* growth medium containing tryptone (1 g liter^−1^) and 16 mM glucose. (Left) Before inoculation (*t* = 0); (right) after 16 h. Cysteine, a component of the medium, is indicated by an asterisk. (C) Growth of *P. bryantii* in Hungate tubes with medium containing 16 mM glucose and tryptone (1 g liter^−1^) (black circles), 16 mM glucose (solid red squares), 4 mM glucose (open red squares), 4 mM glucose and 5 mM fumarate (magenta triangles), tryptone (1 g liter^−1^) (open circles), 20 mM fumarate (open green triangles), and 5 mM fumarate (solid green triangles). Averages and standard deviations for three biological replicates are shown.

QFR is encoded by the *frdCAB* operon (Fig. S1). The complex is composed of FrdA and FrdB, oriented toward the cytoplasm, and the membrane-bound FrdC subunit. This QFR belongs to the type B class of quinol:fumarate oxidoreductases (13). During growth of *P. bryantii* with 16 mM glucose and 1 g/liter tryptone, glucose was completely consumed within 3 h and succinate was formed in parallel, reaching 18 mM in the stationary phase (Fig. 1A). This result clearly suggests that QFR is active and generates succinate from fumarate.

Besides succinate, lactate and malate were identified in the medium after 16 h of growth (Fig. 1B, right). *P. bryantii* produced acetate from glucose (20) as confirmed here (Fig. 1A). This indicated formation of ATP from pyruvate via acetyl coenzyme A (acetyl-CoA) and acetyl phosphate. Peptides and amino acids (from tryptone) were poor growth substrates for *P. bryantii* (Fig. 1C), though tryptone improved growth on glucose slightly (Fig. 1C). With 4 mM glucose, the final optical density at 600 nm (OD_600_) decreased to 0.78 (compared to an OD_600_ of ≥2 with 16 mM glucose). Comparing growth with 4 mM glucose in the absence or presence of 5 mM fumarate revealed no difference in growth rate or yield of *P. bryantii*. When fumarate was the sole carbon source (5 mM or 20 mM), only a slight increase in OD_600_ of 0.05 after 27 h was observed (Fig. 1C). The breakdown of glucose is linked to the production of succinate from endogenously formed fumarate, and this reaction is likely to be catalyzed by QFR. This reaction requires quinol as an electron donor provided by NQR, as described below.

### *P. bryantii* membranes exhibit NADH oxidation and fumarate reduction activities with menaquinone as the electron carrier.

**FIG 2:**
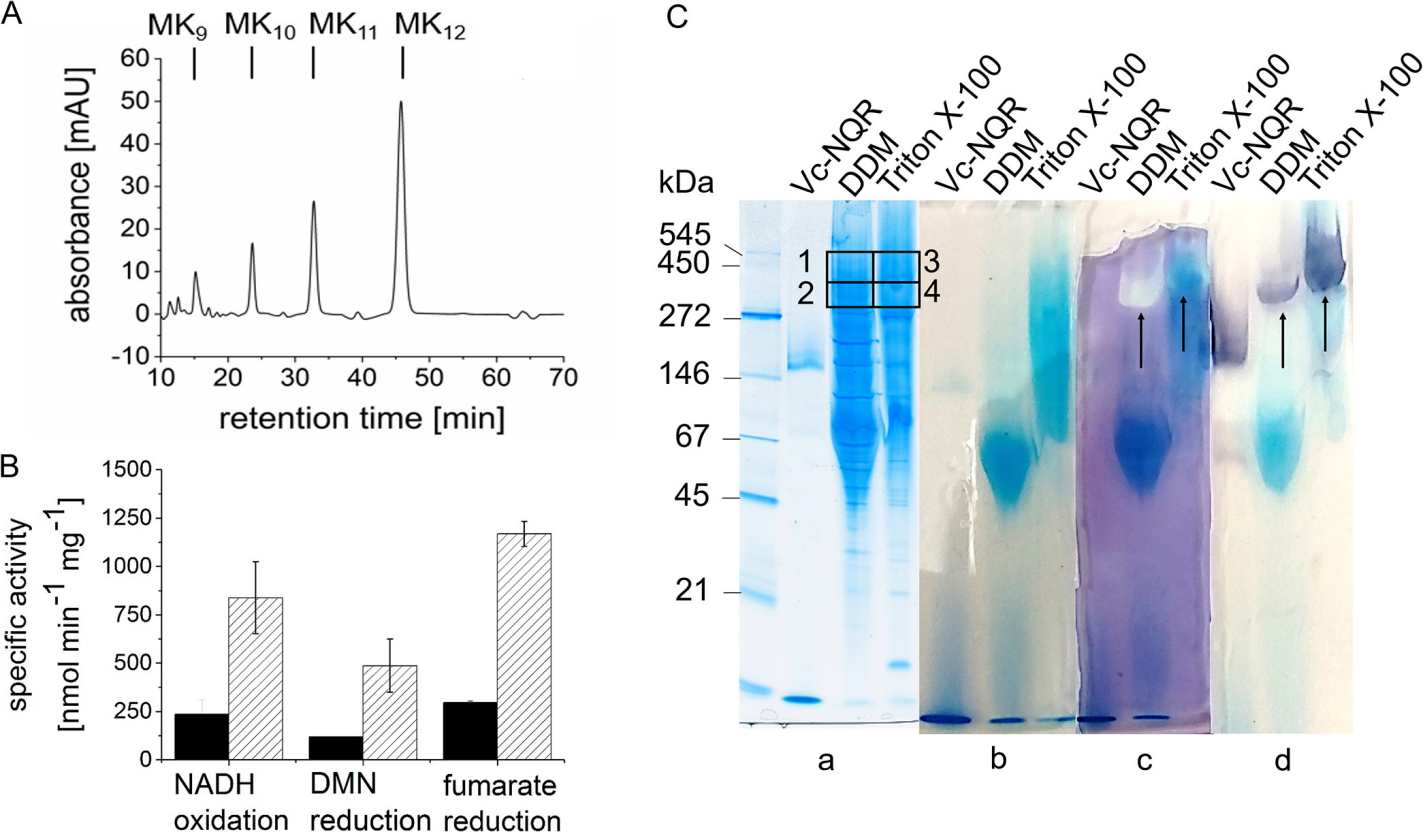
NADH oxidation and fumarate reduction by solubilized membranes from *P. bryantii* with menaquinone as the electron carrier. (A) Quinone composition of membranes from *P. bryantii* analyzed by HPLC. (B) Specific enzyme activities of NADH oxidation, DMN reduction, and fumarate reduction in DDM-solubilized membranes of *P. bryantii*. Solubilized membranes from *P. bryantii* were prepared under oxic (solid bars) or anoxic (hatched bars) conditions. NADH oxidation and DMN reduction were followed simultaneously. Reduction of fumarate was monitored from the oxidation of benzyl viologen at 546 nm in a separate assay. Mean values and standard deviations from three technical replicates are shown. (C) Blue native PAGE of solubilized *P. bryantii* membranes and detection of proteins with NADH dehydrogenase or fumarate reductase activity. Membranes solubilized with 5% Triton X-100 or 2.5% DDM (approximately 70 μg protein per lane) were separated by BN-PAGE with a gradient from 4% to 16% acrylamide. NQR from V. cholerae (4 μg) was used as a control. Each sample was loaded in triplicate, and the BN-PAGE gel was cut into three parts. (a) Coomassie-stained BN gel. Boxes 1 to 4 indicate proteins subjected to tryptic digestion and mass spectrometry analysis. (b) BN gel after run, unstained. (c) BN-PAGE treated to reveal fumarate reductase activity. (d) BN gel treated to reveal NADH dehydrogenase activity. Arrows highlight high-molecular-mass complexes exhibiting both activities.

The NQR from facultative anaerobes such as *Vibrio* spp. was shown to operate with ubiquinone, not menaquinone (12). On the other hand, QFR of *W. succinogenes* reacts with menaquinone (14). The genome of *P. bryantii* harbors genes for menaquinone synthesis (21) but not for ubiquinone synthesis. Quinone extraction followed by high-performance liquid chromatography (HPLC) coupled to mass spectrometry confirmed that *P. bryantii* contains only menaquinones. A typical HPLC profile revealed quinones eluting at 15.2 min, 23.7 min, 32.8 min, and 45.7 min (Fig. 2A) assigned to menaquinones with 9 (MK_9_), 10 (MK_10_), 11 (MK_11_), and 12 (MK_12_) isoprenoid units by mass spectrometry and UV/visible-spectrum (Vis) spectroscopy (Fig. S4). Some extracts also contained traces of MK_13_. MK_12_ is the most abundant quinone in the membrane of *P. bryantii*. Thus, NQR from *P. bryantii* is highly likely to interact with menaquinone. In accord with that notion, 2,3-dimethyl-1,4-naphthoquinone (DMN), a menaquinone derivative with a methyl group replacing the hydrophobic isoprenoid side chain, acted as an *in vitro* electron acceptor for NQR. *P. bryantii* membranes which were prepared under aerobic conditions exhibited NADH oxidation and DMN reduction activities of 150 nmol min^−1 ^mg^−1^ and 70 nmol min^−1 ^mg^−1^, respectively.

Membranes of *P. bryantii* were solubilized with different detergents, such as digitonin, Triton X-100, and *n*-dodecyl β-d-maltoside (DDM), and the activities of solubilized NQR with DMN as the electron acceptor were determined. Digitonin (4%, wt/vol) was not suited for solubilization, since the supernatant after ultracentrifugation contained nearly no protein. With 2% (wt/vol) Triton X-100, nearly half of the total protein of membranes was retrieved in the solubilized supernatant, which exhibited 250 nmol min^−1 ^mg^−1^ NADH oxidation and 80 nmol min^−1 ^mg^−1^ DMN reduction activities. The best results were achieved with DDM (2.5%, wt/vol), solubilizing approximately 75% of the membrane proteins, with 300 nmol min^−1 ^mg^−1^ NADH oxidation and 100 nmol min^−1 ^mg^−1^ DMN reduction activities. In general, preparation of membranes and solubilized membrane proteins from *P. bryantii* under anoxic conditions resulted in increased NQR and QFR activities (Fig. 2B). Specific activities of 300 nmol min^−1 ^mg^−1^ NADH oxidized, 100 nmol min^−1 ^mg^−1^ DMN reduced, and 300 nmol min^−1 ^mg^−1^ fumarate reduced were measured with solubilizates prepared under oxic conditions. Solubilizates prepared under anoxic conditions had specific activities of 850 nmol min^−1 ^mg^−1^ NADH oxidized, 490 nmol min^−1 ^mg^−1^ DMN reduced, and 1,200 nmol min^−1 ^mg^−1^ fumarate reduced (Fig. 2B). The substoichiometric formation of DMNH_2_ from NADH by NQR is likely due to incomplete reduction of quinones by NQR under formation of semiquinones (22), or by electron transfer to O_2_ (23). We previously observed a stimulation of NADH oxidation and quinone reduction activities of *P. bryantii* membranes by Na^+^ (7). In accordance with these findings, specific NADH oxidation and quinone reduction activities of membranes solubilized with DDM decreased by ca. 70% when the Na^+^ concentration in the assays was lowered from 1 mM to 10 μM Na^+^.

### A supercomplex consisting of NQR and QFR in *P. bryantii* membranes.

Aggregation state and protein composition of NQR and QFR in DDM and Triton X-100 solubilizates were studied by blue native PAGE (BN-PAGE) followed by in-gel activity staining and mass spectrometry. The apparent masses of protein complexes were estimated from a comparison with standards covering the range from 545 to 21 kDa and with purified NQR from Vibrio cholerae with an apparent mass of 220 kDa (11). Purified NQR from V. cholerae and DDM and Triton X-100 solubilizates were separated by BN-PAGE in triplicate, and separate staining was performed with Coomassie blue to stain all proteins (Fig. 2C, panel a), with NADH plus nitroblue tetrazolium chloride (NBT) to stain proteins exhibiting NADH dehydrogenase activity (Fig. 2C, panel d), and with reduced benzyl viologen (BV_red_) plus fumarate to stain proteins exhibiting fumarate reductase activity (Fig. 2C, panel c). A control shows NQR, DDM, and Triton X-100 solubilizates before staining (Fig. 2C, panel b). Purified NQR complex from V. cholerae comprising six subunits (NqrABCDEF) migrated as single band at an apparent mass slightly above 146 kDa, as detected by Coomassie staining (Fig. 2C, panel a) and by NADH oxidation activity of subunit NqrF (Fig. 2C, panel d). Much larger protein complexes exhibiting NADH dehydrogenase activity were detected in membranes solubilized with DDM or Triton X-100, with one prominent complex migrating slightly above the 272-kDa marker (DDM) and at least two complexes slightly above 272 kDa and around 500 kDa (Triton X-100). This suggested that NqrF was part of a supercomplex. The prominent protein bands above 272 kDa exhibited both NADH oxidation and fumarate reductase activities (Fig. 2C, panels c and d), indicating a supercomplex composed of NQR and QFR. This supercomplex was observed in 5 biological replicates. To study the protein composition of supercomplexes, Coomassie-stained protein bands migrating at molecular masses similar to those of the bands detected by enzyme activity staining were excised from the gel (Fig. 2C, panel a, boxes 1 to 4) and subjected to tryptic digestion and mass spectrometry.

**FIG 3:**
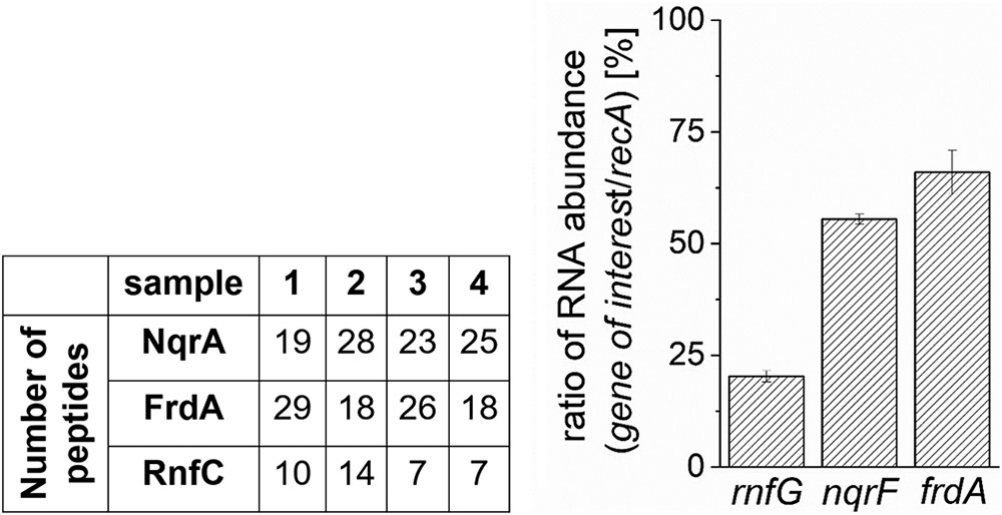
Relative abundance of NQR, QFR, and RNF in *P. bryantii*. (Left) Number of unique peptides from NqrA, FrdA, and RnfC subunits identified by mass-spectrometric analyses of samples 1 to 4, obtained from Coomassie-stained BN-PAGE gels (Fig. 2C, panel a). (Right) Abundance of *rnfG*, *nqrF*, and *frdA* transcripts in relation to abundance of *recA* (100%). Samples from two biological replicates were measured in three technical replicates each.

Besides NQR and QFR, *P. bryantii* contains genes coding for the *Rhodobacter* nitrogen fixation (RNF) complex, the electrogenic ferredoxin:NAD^+^ oxidoreductase (24), and an 11-subunit complex with putative electron transfer and cation transport activity (8, 25) (Fig. S1). Mass-spectrometric data from samples 1 to 4 (Fig. 2C) were analyzed with respect to the presence of the subunits of membrane-bound complexes. Large amounts of unique peptides were found for subunits NqrA from NQR and FrdA from QFR (Fig. 3). We also detected smaller amounts of peptides indicating the presence of subunits from RNF and the 11-subunit complex. Subunits NqrA, FrdA, and RnfC are large hydrophilic subunits (Table S1) which are readily detected. Results of an *in silico* tryptic digestion of NQR, QFR, and RNF are presented in Table S2. Peptides from NQR, QFR, and RNF subunits identified by mass spectrometry (Table S3), and the complete set of mass-spectroscopic data (Table S4) are also reported. The comigration on BN-PAGE at an apparent molecular mass higher than that of the NQR complex isolated from V. cholerae (Fig. 2C) suggested tight interaction of NQR and QFR in a sodium-NQR fumarate reductase (SNFR) supercomplex. These high-molecular-mass protein bands exhibited both NADH oxidation and fumarate reduction activities. Samples 2 and 4 (Fig. 2C) migrating at an apparent mass above 272 kDa could represent a supercomplex comprising one NQR complex and one QFR complex (calculated mass, 330 kDa). Protein bands exhibiting NADH oxidation and fumarate reduction activities migrating at 450 kDa (Fig. 2C, samples 1 and 3) could represent aggregates of NQR and QFR at different ratios or bound to other proteins. Note that QFRs may form dimers as observed in three-dimensional (3D) structures of QFRs from Wolinella succinogenes and Escherichia coli (26, 27).

Furthermore, gene expression of the *rnfG*, *nqrF* and *frdA* genes of *P. bryantii* was analyzed by reverse transcription-quantitative PCR (RT-qPCR) (Fig. 3) in two biological replicates. As a reference, expression of the housekeeping gene *recA* was analyzed. The transcript level of *rnfG* reached only 19% of the level of *recA*. With *nqrF* and *frdA*, 54% and 66% of the transcript level of *recA* were observed, respectively. The abundance of bacterial proteins is mainly determined by the transcript level (28). Expression levels of *nqrF* and *frdA* were similar and more abundant than *rnfG* transcripts, in accord with the observed prevalence of peptides from NQR and QFR subunits.

### Properties of the SNFR supercomplex.

**FIG 4:**
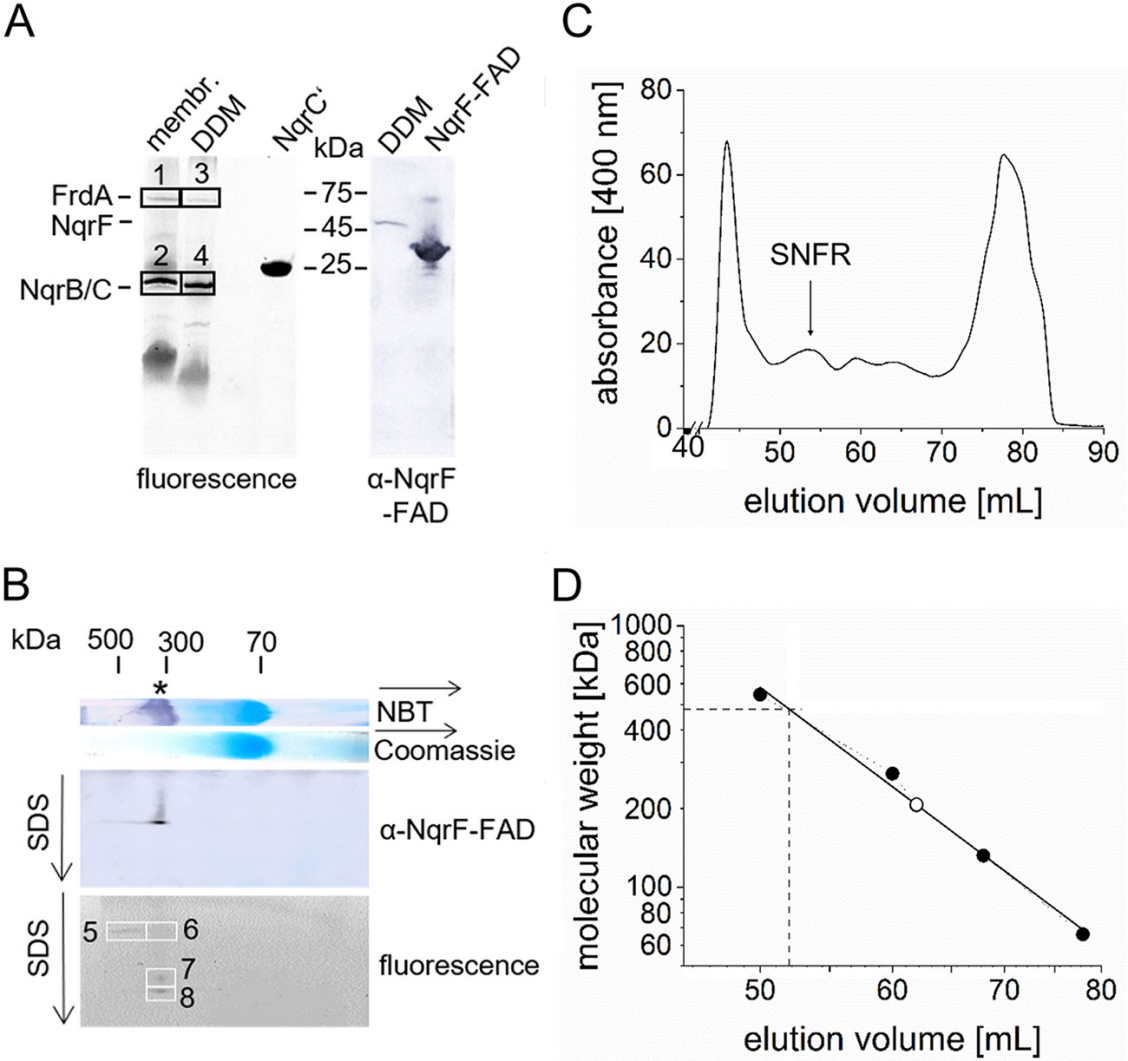
Characterization of the SNFR supercomplex from *P. bryantii*. (A) Membranes (100 μg) and membranes solubilized with DDM (100 μg) were separated by SDS-PAGE to detect flavinylated proteins (left, with 2 μg of truncated NqrC′ protein as the positive control). (Right) Western blot of DDM solubilizates (100 μg) and the FAD-binding domain of subunit NqrF (NqrF-FAD; 10 μg) using anti-NqrF-FAD domain antibodies. (B) 2D BN/SDS-PAGE of DDM solubilizates (70 μg). The lane from a BN-PAGE gel stained with Coomassie was placed on top of the SDS-PAGE gel. NADH dehydrogenase activity stain (NBT) indicated the position of the SNFR supercomplex (*). Flavinylated proteins in the SDS-PAGE were detected by in-gel fluorography. Afterward, immunostaining with anti-NqrF-FAD domain antibodies was performed. Boxes 1 to 8 indicate the positions of fluorescent protein bands subjected to proteolysis and mass spectrometry for identification of subunits. (C) SNFR in DDM solubilizates separated by size exclusion chromatography. (D) The molecular weight of SNFR was estimated from a calibration with protein standards (66 kDa, 132 kDa, 220 kDa, 272 kDa, and 545 kDa).

NQR and QFR subunits of the SNFR supercomplex in solubilizates were identified by in-gel fluorography and immune staining. Subunits NqrB (41.7 kDa), NqrC (23.2 kDa) and FrdA (73.6 kDa) exhibit fluorescence in the denatured state, due to covalently bound flavins (29, 30). Prominent fluorescent bands slightly below 25 kDa were observed both in native and in DDM-solubilized membranes from *P. bryantii* after separation by SDS-PAGE (Fig. 4A, left). These bands corresponded to subunits NqrB and NqrC, which comigrated under these conditions (7, 31). Due to their hydrophobicity, they migrate below NqrC′, a truncated version of NqrC from V. cholerae (32). NqrC′ lacks the hydrophobic transmembrane helix but retains the covalently attached FMN, giving rise to a fluorescent band with a molecular mass of 25 kDa (Fig. 4A, left). A protein band with weak fluorescence intensity at about 75 kDa in native and solubilized membranes was assigned to FrdA with its covalently attached FAD (Fig. 4A, left). NqrB, NqrC, and FrdA were identified in the fluorescent protein bands (Fig. 4A, boxes 1 to 4) by mass-spectrometric analyses. A complete list of identified peptides is provided in the electronic supplemental material (Table S5).

In order to identify the NqrF subunit in solubilized *P. bryantii* membranes, antibodies were raised against the purified FAD domain of *P. bryantii* NqrF. An immune-reactive band around 35 kDa was observed with the purified FAD domain of NqrF, in accordance with its calculated molecular mass (33.5 kDa) (Fig. 4A, right). As expected, solubilized membranes exhibited an immune-reactive band at about 45 kDa, which was assigned to full-length NqrF with a calculated molecular mass of 47.1 kDa (Fig. 4A, right).

To study the stability of the SNFR supercomplex solubilized from *P. bryantii* membranes with DDM, second-dimension SDS-PAGE was performed after first-dimension BN-PAGE. The SNFR with an apparent mass of 330 kDa identified by NADH dehydrogenase activity stain in BN-PAGE dissociated into three prominent complexes (bands 6, 7, and 8 in Fig. 4B). Band 6 exhibited cross-reactivity with anti-NqrF-FAD domain antibodies. Bands 5, 6, 7, and 8 contained covalently attached flavins, indicating the presence of FrdA, NqrB, and NqrC subunits. Their prevalence was confirmed by mass-spectrometric analyses of peptides derived from band 6 (Table S6), with FrdA (22 peptides), FrdB (4 peptides), NqrA (22 peptides), NqrF (7 peptides), NqrC (6 peptides), and NqrB (3 peptides). FrdC, NqrD, and NqrE were not detected, but note that these small, hydrophobic proteins (Table S1) are difficult to detect by the mass spectrometry method used here. Bands 7 and 8 migrated at lower apparent masses in the second dimension SDS-PAGE, most likely due to the lack of NqrF and NqrB, which were not detected by mass spectrometry. Bands 7 and 8 exhibited very similar compositions (Table S6), with FrdA (8 peptides), FrdB (1 to 5 peptides), NqrA (3 peptides), and NqrC (3 peptides). In the DDM solubilizates, the minor fraction of 500 kDa SNFR observed on BN-PAGE (Fig. 2C) was also detected by second-dimension SDS-PAGE (band 5 in Fig. 4B), containing peptides from FrdA (27 peptides), FrdB (10 peptides), NqrA (5 peptides), NqrF (4 peptides), NqrC (1 peptide), and NqrB (1 peptide). Peptides derived from other membrane-bound and soluble proteins were also detected (Table S7).

We conclude that the SNFR supercomplex does not readily dissociate despite the presence of SDS, indicating tight interactions between NQR and QFR. Size exclusion chromatography of the DDM solubilizate confirmed this notion (Fig. 4C). A large complex containing subunits NqrF, NqrB/C, and FrdA and heme *b* (Fig. S5) eluting at 55 ml represented SNFR with an apparent molecular weight of 480 kDa (Fig. 4D).

### Intramolecular electron transfer in the SNFR supercomplex.

Electron transfer between substrates and redox centers of SNFR was followed by recording UV/Vis difference spectra. The redox state of FrdC hemes *b* indicated the degree of reduction of QFR. Reduced hemes *b* have absorption maxima at 560 nm, 527 nm, and 427 nm. In their oxidized state, they exhibit a characteristic absorption maximum at 410 nm. QFR and NQR contain flavins, which can be detected by a decrease of absorbance at 448 nm upon reduction. First, these redox cofactors of SNFR in *P. bryantii* membranes were identified after reduction with a large excess of dithionite. A cuvette containing air-oxidized membranes in 20 mM potassium phosphate buffer pH 7.5 was placed in beam 1 of the double-beam photometer. A second cuvette containing dithionite-reduced membranes was analyzed in beam 2.

**FIG 5:**
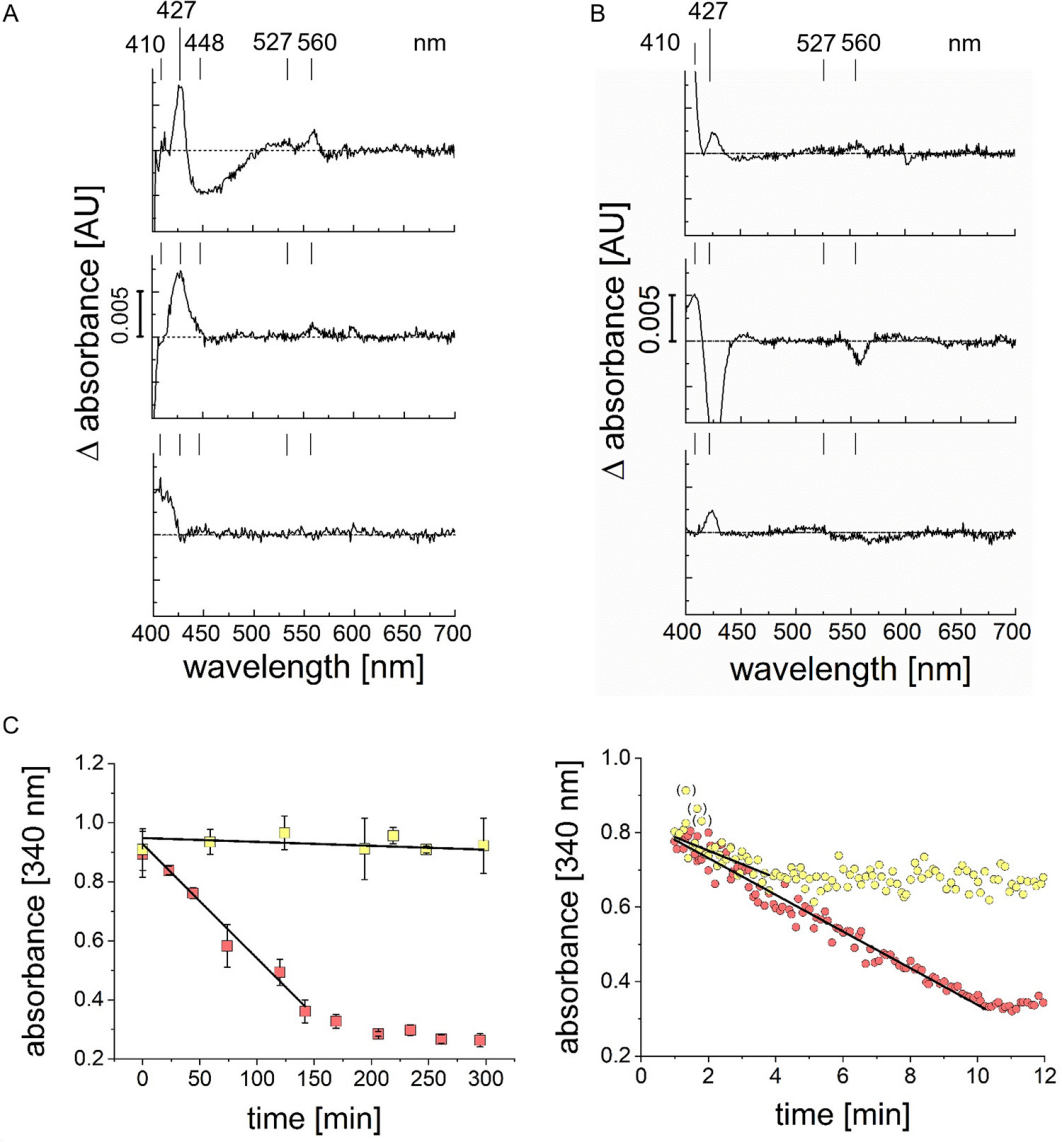
Membrane-bound redox centers and NADH:fumarate oxidoreduction activity of *P. bryantii*. Vis difference spectra of membranes (0.5 mg protein/ml) (A) and Triton X-100-solubilized membranes (0.5 mg protein/ml) (B) with signatures of reduced heme *b* (427, 527, and 560 nm), oxidized heme *b* (410 nm), and reduced flavin (448 nm). (A) Traces, from top to bottom: [dithionite reduced] minus [air oxidized], [NADH reduced] minus [air oxidized], [NADH reduced] minus [NADH reduced with added fumarate]. (B) Traces, from top to bottom: [NADH reduced] minus [air oxidized], [NADH reduced] minus [NADH reduced with added fumarate], [succinate reduced] minus [air oxidized]. (C) Oxidation of NADH (initial concentration, 200 μM) by membranes (0.1 mg protein). (Left) No electron acceptor added (yellow squares) or 10 mM fumarate added (red squares). (Right) DMN (65 μM) (yellow circles) or with 65 μM DMN and 10 mM fumarate (red circles) added. Typical traces from at least 3 replicates are presented. In panel C (left), means and standard deviations (*n* = 3) are shown.

Figure 5A (top) shows the difference spectrum of the dithionite-reduced minus the air-oxidized membranes. Three maxima at 560 nm, 527 nm, and 427 nm were observed, with a minimum at 448 nm. These were assigned to fully reduced hemes *b* and flavins, respectively. We asked if NADH reduces the hemes *b* of the QFR in *P. bryantii* membranes under participation of NQR as the electron entry site. Two quartz cuvettes were filled with aliquots of air-oxidized membranes of *P. bryantii*. To the aliquot in beam 2, 125 μM NADH were added, and the difference spectrum of NADH-reduced minus air-oxidized membranes was recorded. Peaks at 427 and 560 nm revealed partial reduction of hemes *b* of QFR, indicating electron transfer from NADH via NQR and quinol to QFR (Fig. 5A, middle). If these hemes *b* are redox centers of QFR, their reoxidation with fumarate should be possible. To test this assumption, membranes were allowed to react with 125 μM NADH, one aliquot was mixed with 12.5 mM fumarate, and the difference spectrum was recorded (Fig. 5A, bottom). The shift in maximum peak to 410 nm indicated the presence of oxidized hemes *b*, demonstrating reoxidation of QFR by fumarate. These results demonstrated electron transfer from NADH to fumarate by NQR and QFR in *P. bryantii* membranes.

Electron transfer was also tested after solubilization of membranes with Triton X-100. Figure 5B (top) shows the difference spectrum of the NADH-reduced minus the air-oxidized solubilized membranes. Here, the prominent maximum of reduced heme *b* at 427 nm was detected. Figure 5B (middle) depicts the difference spectrum of the NADH-reduced solubilizates mixed with fumarate, minus the NADH-reduced solubilizate. Three minima at 428 nm, 527 nm, and 560 nm were detected, indicating reoxidation of the hemes *b* of QFR by fumarate. Furthermore, a maximum at 410 nm appeared which is characteristic for oxidized hemes *b*. These findings are in accord with NADH:fumarate oxidoreduction by the combined action of NQR and QFR in the SNFR supercomplex. QFR catalyzes succinate:quinone oxidoreduction under participation of hemes *b* (33). Air-oxidized Triton X-100-solubilized membranes (beam 1) were compared with an aliquot treated with 12.5 mM succinate (beam 2). In the difference spectrum of succinate-reduced minus air-oxidized solubilized membranes (Fig. 5B, bottom), a typical maximum of reduced heme *b* at 427 nm was identified. Comparing the change in absorbance units (ΔAU) of NADH- or succinate-reduced solubilized membranes revealed similar intensities (∼0.0025 AU), suggesting that a similar proportion of hemes *b* in QFR underwent reduction.

Rate measurements of NADH oxidation by *P. bryantii* membranes with DMN and/or fumarate as electron acceptors showed only residual activity in the absence of both DMN and fumarate (0.2 nmol min^−1 ^mg^−1^) (Fig. 5C, left). Adding fumarate increased the rates to ∼10 nmol min^−1 ^mg^−1^ and after approximately 150 min nearly all NADH was consumed (150 nmol from an initial 200 nmol) (Fig. 5C, left). With DMN as the electron acceptor, NADH oxidation activity increased to 150 nmol min^−1 ^mg^−1^ (Fig. 5C, right), indicating that the amount of endogenous menaquinone limits electron transfer *in vitro*. Interestingly, after ∼5 min and a consumption of 75 nmol NADH, the NADH concentration did not further decrease, reflecting an equilibrium of DMN/DMNH_2_ and NAD^+^/NADH. Highest NADH oxidation activity was observed in the presence of both DMN and fumarate (200 nmol min^−1 ^mg^−1^) (Fig. 5C, right). After 10 min, NADH was completely oxidized. We conclude that fumarate acts as an electron acceptor for the SNFR supercomplex in its native membrane environment. Menaquinone acts as the electron carrier between NQR and QFR. Short-chain menaquinones such as DMN stimulated overall SNFR activity 20-fold, suggesting exchange of SNFR-bound and free menaquinone, as previously reported for other supercomplexes (34).

### Succinate formation by the SNFR supercomplex.

**FIG 6:**
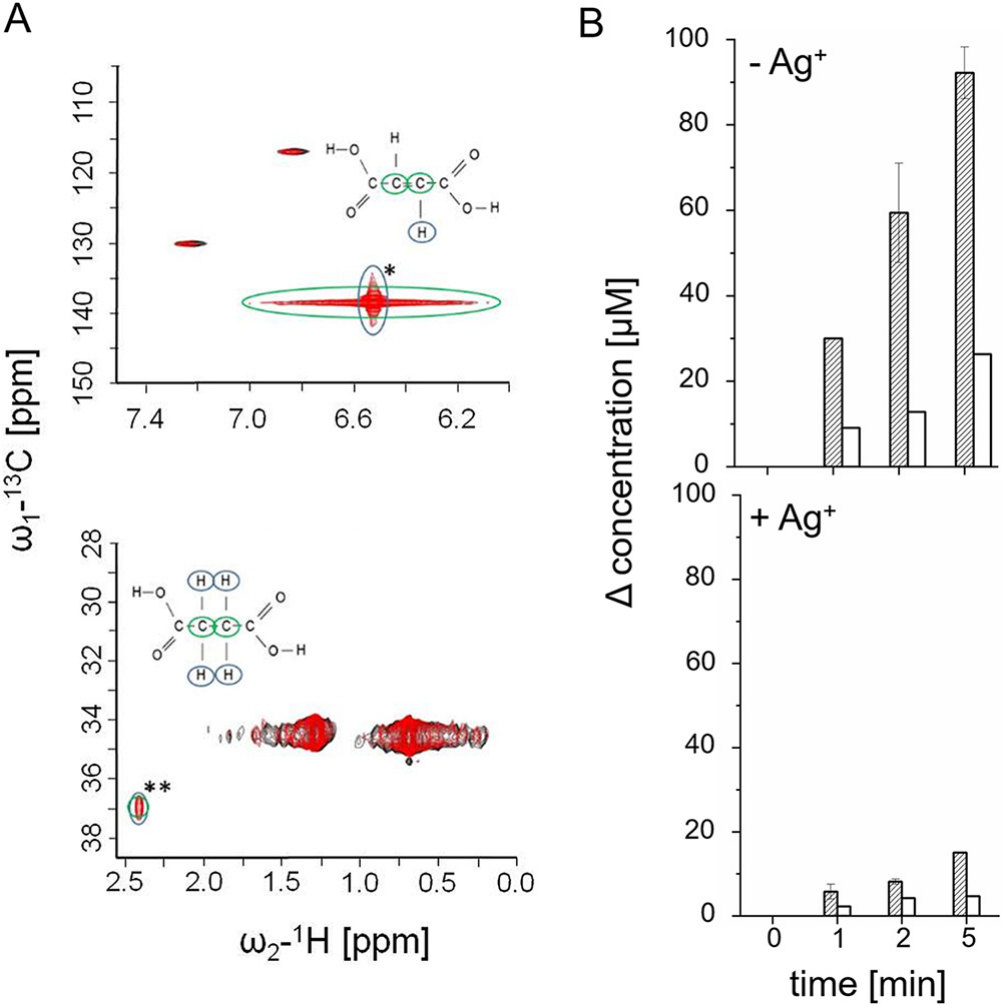
Succinate formation by the SNFR supercomplex from *P. bryantii*. (A) Membranes (100 μg) were allowed to react with NADH, DMN, and fumarate for 12 min, and 2D ^1^H and ^13^C NMR spectra were recorded (red traces). In the control reaction (black traces), fumarate was omitted. (Top) Fumarate peak in 2D ^1^H and ^13^C NMR spectrum; (bottom) succinate peak in 2D ^1^H and ^13^C NMR spectrum. Typical resonances for fumaric acid (*) and succinic acid (**) are indicated. Resonance assigned to carbon atoms (green) and hydrogen atoms (blue) are highlighted. (B) NADH oxidation by DDM solubilizate (100 μg) in the presence of DMN and fumarate was followed at 340 nm. At indicated times, aliquots were subjected to 1D ^1^H NMR to determine succinate concentrations. NADH consumed (hatched bars) and succinate formed (open bars) are presented as the concentration difference at indicated time and at the start of the reaction. The reaction was performed in the absence (top) or presence (bottom) of 3 μM AgNO_3_. Averages and standard deviations for two technical replicates are shown.

*P. bryantii* membranes catalyzed the complete oxidation of NADH (200 μM) with an excess of fumarate (10 mM) in the presence of only 65 μM DMN (Fig. 5C), indicating electron transfer to fumarate. Using nuclear magnetic resonance (NMR) spectroscopy, we demonstrated stoichiometric formation of succinate in the reaction assay containing membranes, NADH, DMN, and fumarate. After completion of the reaction (12 min), aliquots were retrieved for 1D ^1^H, 2D ^1^H, and ^13^C NMR measurements. The assay mixture devoid of fumarate (but with NADH and DMN) served as a control. Figure 6A depicts the 2D ^1^H and ^13^C NMR spectra, highlighting the typical ranges of fumarate (top) and succinate (bottom). The dicarboxylic acids are distinguished by the chemical shift of C-3 and C-4 carbon atoms and their bound H atoms. The complete reaction mix revealed resonances assigned to succinic acid, which were not detected in the control reaction devoid of fumarate. Quantification of the ^1^H NMR signal showed that the oxidation of 200 μM NADH led to the formation of 170 ± 0.02 μM succinic acid (*n* = 3), indicating transfer of electrons from NADH to fumarate. We followed time-dependent formation of succinate with NADH as the electron donor using DDM-solubilized membranes and studied the effect of Ag^+^, a specific inhibitor of NQR (35, 36) (Fig. 6B). At each time point (1, 2, and 5 min after start of the reaction), the concentrations of NADH and succinate were determined. Per mole of succinate, approximately 3 mol NADH was consumed, in accord with ratios of NADH oxidation and quinone reduction activities observed with DDM solubilizates (Fig. 6B, top). In the presence of Ag^+^, succinate formation was inhibited by at least 80%, demonstrating that fumarate reduction in *P. bryantii* membranes required active NQR (Fig. 6B, bottom).

### An electrochemical sodium gradient in *P. bryantii*.

**FIG 7:**
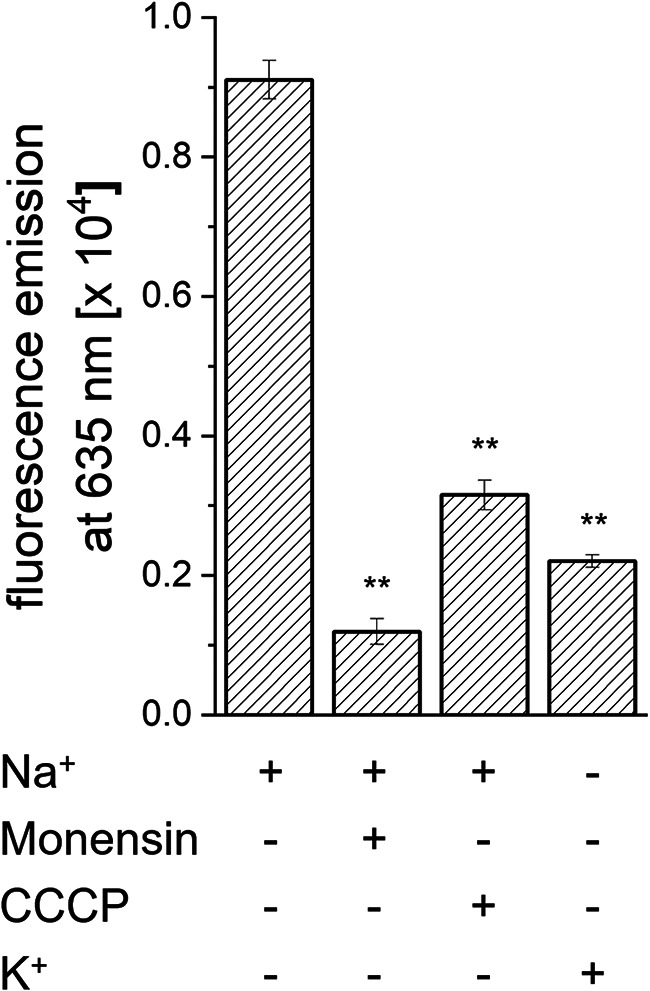
Electrochemical Na^+^ gradient in *P. bryantii*. The intensity of fluorescence emission is a measure for the membrane potential established by cells. The fluorescence emission of the fluorophore without added cells (control) was subtracted. The ionophores monensin and carbonyl cyanide *m*-chlorophenylhydrazone (CCCP) (each at 2.5 μM) were added as indicated. Mean values and averages from three technical replicates are shown. Asterisks indicate significant differences from cells incubated with Na^+^ in the absence of ionophore (*P* < 0.05).

We studied the formation of a membrane potential (ΔΨ) by *P. bryantii* using the dye DiOC_2_ (3,3′-diethyloxacarbocyanine iodide). The uptake of DiOC_2_ by cells is promoted by ΔΨ (inside negative), and cells with a higher ΔΨ exhibit increased red fluorescence. The highest ΔΨ was observed in the presence of 155 mM Na^+^. Fluorescence emission decreased by ca. 80% when Na^+^ was replaced by K^+^ (Fig. 7). Incubation of Na^+^-treated cells with the Na^+^-specific ionophore monensin resulted in a drastic decrease in transmembrane voltage by ca. 90%. With the protonophore carbonyl cyanide 3-chlorophenylhydrazone (CCCP), ΔΨ was decreased by ca. 70%. These results demonstrate that *P. bryantii* established an electrochemical Na^+^ gradient, suggesting redox-driven Na^+^ transport by the NQR in *P. bryantii*. Besides the Na^+^ gradient, *P. bryantii* maintained an electrochemical H^+^ gradient, as shown by partial dissipation of ΔΨ in the presence of a protonophore.

## DISCUSSION

**FIG 8:**
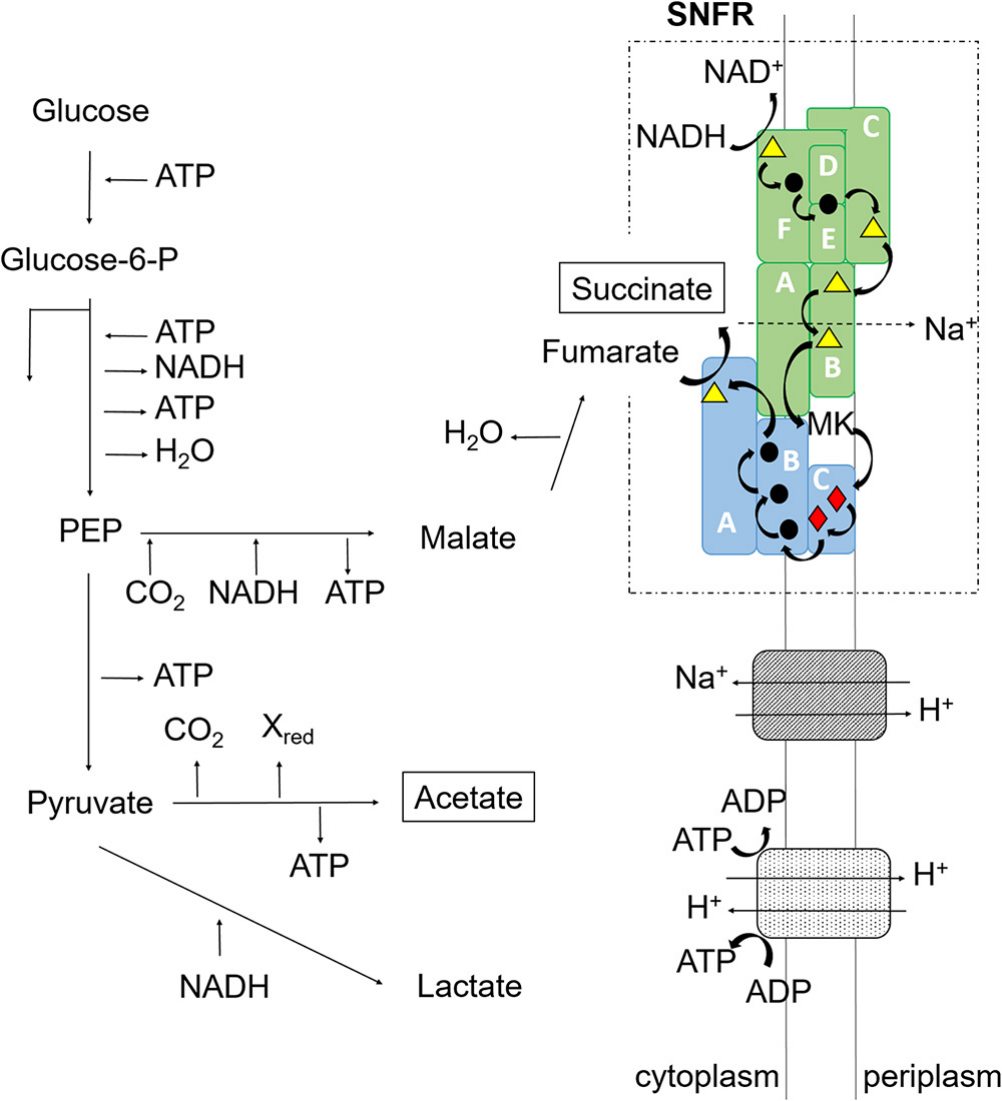
The SNFR supercomplex represents the major charge-separating module in *P. bryantii.* Major end products are shown in black boxes. Blue, fumarate reductase (QFR); green, Na^+^-translocating NADH:quinone oxidoreductase (NQR); hatching, Na^+^/H^+^ antiporter; stippling, F_1_F_o_ ATPase/ATP synthase. Subunits of NQR (lettered A to F) and QFR (lettered A to C) are indicated. Colored symbols in the protein complexes represent cofactors. Yellow triangle, flavin; black circle, iron-sulfur center; red diamond, heme *b*. NQR and QFR form a sodium-translocating NADH:fumarate oxidoreductase (SNFR) supercomplex (dashed box).

In this study, evidence for a sodium-translocating NADH:fumarate oxidoreductase complex (SNFR) in *P. bryantii* is presented. SNFR is a membrane-bound supercomplex composed of the Na^+^-translocating NADH:quinone oxidoreductase (NQR) and the fumarate reductase (QFR). SNFR represents an important functional module, which most likely generates a sodium-motive force in *P. bryantii*. This supercomplex intimately connects NADH-driven sodium ion translocation to an important step in sugar degradation by *P. bryantii*, namely, the formation of succinate, which is excreted as an end product (8, 37) (Fig. 8). Menaquinone acts as an electron carrier between NQR and QFR within the SNFR supercomplex. QFR of *P. bryantii* is closely related to the *W. succinogenes* enzyme and is therefore thought not to contribute to the generation of an electrochemical potential. It has been shown for the QFR of *W. succinogenes* that the transmembrane electron transfer from quinol, located on the periplasmic side, via the heme groups to the fumarate reduction site, located on the cytoplasmic side, is coupled to compensatory proton flux from the periplasm into the cytoplasm (38). Thus, the overall reaction of *W. succinogenes* QFR is electroneutral. If *P. bryantii* and *W. succinogenes* QFR share the same architecture, only NQR contributes to membrane potential formation in the SNFR complex. In SNFR, a donor:quinone dehydrogenase (NQR) is coupled to a quinol:acceptor reductase (QFR) in a simple electron transfer chain that produces a sodium-motive force. This module is reminiscent of the formate (or H_2_):fumarate oxidoreductase system in *W. succinogenes*, where the electrogenic formate (H_2_):quinone oxidoreductase provides quinol to the nonelectrogenic quinol:fumarate oxidoreductase (QFR) (39). With the help of SNFR, the disposal of redox equivalents by succinate formation, as well as the regeneration of NAD^+^ required for the initial breakdown of glucose, is coupled to the buildup of a sodium-motive force. Thus, SNFR increases the energy yield during growth of *P. bryantii* on glucose.

Addition of fumarate did not improve growth of *P. bryantii* with glucose as the carbon source, indicating that *P. bryantii* did not benefit from surplus electron acceptor under the given growth conditions. Possible explanations are (i) the lack of fumarate uptake systems and (ii) the endogenous supply of fumarate produced during the degradation of glucose. *P. bryantii* harbors a gene related to *dcuB* (UniProt accession number D8DV98), coding for a putative succinate:fumarate antiporter (40). Also, two genes coding for putative Na^+^-coupled dicarboxylate transporters are found (UniProt accession numbers A0A1H9DU78 and D8DTN2) (41). This suggests that the utilization of exogenous fumarate by *P. bryantii* is not limited by transport. Rather, one would suspect that the rate of fumarate formation from glucose is high, resulting in cytoplasmic fumarate concentrations that are saturating for the SNFR supercomplex. Notably, we observed roughly equimolar conversion of glucose (16 mM) to succinate (18 mM). This is in marked contrast to mixed-acid fermentation in E. coli, where ca. 0.2 mol succinate is formed from 1 mol glucose (40). In *P. bryantii*, phosphoenolpyruvate (PEP) is carboxylated by PEP-carboxykinase, yielding nucleotide (ATP or GTP) and oxaloacetate. Oxaloacetate is reduced to malate, and the conversion of malate by fumarase yields fumarate. This pathway to fumarate, and the subsequent reduction to succinate by the SNFR, is an important metabolic route leading to the generation of an electrochemical sodium gradient in *P. bryantii* (Fig. 8). Considering critical, conserved residues in the cation binding site (42), the F_1_F_o_ synthase of *P. bryantii* is a proton- rather than a sodium-dependent enzyme, but the bioenergetic sodium and proton cycles are connected by the putative Na^+^/H^+^ antiporters NhaC and NhaD (UniProt accession numbers D8DUX6 and D8DYG5) of *P. bryantii*. Hence, the sodium-motive force established by SNFR will promote ATP synthesis (Fig. 8).

In the past, feeding experiments with cattle were conducted to test if fumarate addition to the diet reduces methane production. Several bacterial species in the rumen are considered to reduce fumarate under oxidation of hydrogen, which also acts as an electron donor for methanogens. Providing fumarate was expected to diminish hydrogen levels, resulting in overall lower methane production. This hypothesis has not yet been unequivocally confirmed *in vivo* (43) or *in vitro* with batch cultures (4). A metaproteomic survey of the rumen revealed up to 12 other *Prevotella* species utilizing NQR and QFR (7). The rumen microbial community is dominated by *Prevotella* spp. Our finding that *P. bryantii* does not benefit from exogenous fumarate at high glucose concentrations offers a rationale for previous feeding experiments.

## MATERIAL AND METHODS

### Bacterial strains and growth conditions.

*P. bryantii* B_1_4 was grown at 39°C in a synthetic medium composed of 1% tryptone (wt/vol), d-glucose (as indicated), 50 mM NaHCO_3_, 15% (vol/vol) mineral solution 1 (17 mM K_2_HPO_4_), 15% (vol/vol) mineral solution 2 [17 mM KH_2_PO_4_, 45 mM (NH_4_)_2_SO_4_, 100 mM NaCl, 5 mM MgSO_4_, 5.4 mM CaCl_2_], 0.44 μM resazurin sodium salt, and short-chain fatty acids (vol/vol): 0.17% acetic acid, 0.01% *n*-valeric acid, 0.01% isovaleric acid, 0.03% *n*-butyric acid, 0.01% isobutyric acid, and 0.06% propionic acid. Medium was prepared and boiled. Immediately after boiling, 8 mM l-cysteine HCl was added. While cooling on ice, the medium was gassed with CO_2_ continuously. The pH of the medium was adjusted to 7.5 using NaOH. The medium was used to fill Hungate tubes (7 ml) or serum bottles (100 ml or 1 liter) which were flushed with CO_2_. Tubes and bottles were sealed gas tight with rubber stoppers and were autoclaved. After autoclaving, 1.5% (vol/vol) of a sterile vitamin solution was added to each tube. The vitamin solution contained 105 μM hemin, 250 μM menadione, 8.5 μM folic acid, 420 μM thiamine hydrochloride, 372 μM riboflavin, 1.2 mM nicotinamide, 580 μM pyridoxamine dihydrochloride, 296 μM calcium pantothenate, 51 μM aminobenzoic acid, 14 μM biotin, and 0.3 μM cyanocobalamin. Medium was inoculated with 5% (vol/vol) of an active culture or glycerol stock. To follow glucose consumption and succinate production during growth of *P. bryantii*, cells were cultivated in 1 liter medium at 39°C with stirring. Samples for determination of optical density, glucose and succinate were taken with a syringe. Growth experiments with glucose, tryptone, or fumarate (added separately or in combination, as indicated) were performed in triplicate using Hungate tubes. The optical density of the cultures at 600 nm was determined with a cell density meter (WPA Biowave CO8000) allowing measurements up to an OD_600_ of 2. If tryptone was omitted, 10 mM NH_4_Cl and 0.36 μM methionine were added to the medium.

### Membrane potential.

Δψ established by *P. bryantii* cells was estimated using the BacLight bacterial membrane potential kit (Invitrogen) and a Tecan Infinite F200 Pro plate reader (44). *P. bryantii* cells were cultivated in Hungate tubes until an OD_600_ of 0.8 was reached. The following steps were performed in the anaerobic chamber. Cells were harvested, diluted in sodium buffer (10 mM sodium phosphate [pH 7.4], 145 mM NaCl) or potassium buffer (10 mM potassium phosphate [pH 7.4], 145 mM KCl) and resuspended in the corresponding buffer to yield a cell suspension with an OD_600_ of 0.25. The residual Na^+^ concentration of the potassium buffer was ∼320 μM. Aliquots (800 μl) were centrifuged (16,000 × *g*, 5 min), and cells were washed twice in the corresponding buffer. Cell suspensions were mixed with CCCP or monensin (final concentration, 2.5 μM) as indicated. After incubation for 10 min (20°C), the fluorescent dye 3,3′-diethyloxacarbocyanine iodide (DiOC_2_; 15 μM) was added, and incubation was continued for 60 min in the dark. Outside the glove box, three aliquots (200 μl) of each sample were applied to a black, flat-bottom 96-well plate (polystyrene; 4titude). To determine red fluorescence intensities, excitation was set to 480 nm (bandwidth, 20 nm) and emission to 635 nm (bandwidth, 35 nm; gain, 117). Fluorescence emission intensities were in the linear range of the fluorescence detector. Fluorescence intensities of buffer with dye and of cell suspensions were determined for background corrections. As expected, an increase in red fluorescence intensity indicating a transmembrane potential was accompanied with a decrease in green fluorescence intensity monitored in parallel (emission, 535 nm; bandwidth, 25 nm; gain, 107).

### Expression and purification of the NqrF-FAD domain of *P. bryantii* NQR.

The FAD domain of subunit NqrF of *P. bryantii* NQR was purified as described for the FAD domain of V. cholerae NqrF (45), modified as follows. The coding fragment of the NqrF-FAD domain (amino acids 129 to 422), comprising an N-terminal His_6_ tag followed by a human rhinovirus (HRV)-3C protease cleavage site, was cloned into pET15b (Fig. S7), yielding plasmid pPbyF1. Gene synthesis, cloning, and transformation of E. coli Tuner(DE3) were performed by GenScript (USA). E. coli pPbyF1 was grown in medium (1.6% [wt/vol] tryptone, 1% [wt/vol] yeast extract, 0.5% [wt/vol] NaCl, 10 mM glucose, 200 μg/ml ampicillin) at 37°C to the exponential phase, and gene expression was induced by addition of 0.5 mM isopropyl-1-thio-β-d-galactopyranoside (IPTG). After 16 h of induction at 20°C, cells were harvested by centrifugation and washed once with 10 mM Tris-HCl (pH 7.5), 300 mM NaCl. The cells were disrupted in a continuous cell lysis system at 20,000 lb/in^2^ (Emulsiflex C3; Avestin). The FAD domain was purified by nickel affinity chromatography (46), using buffer A (50 mM sodium phosphate [pH 8.0], 300 mM NaCl) for loading, buffer A with 5 mM imidazole for washing, and buffer A with 400 mM imidazole for elution. Thirty-seven units of HRV-3C protease comprising a His_6_ tag (47) per mg FAD domain was added, and the combined proteins were dialyzed against buffer A overnight at 4°C. After dialysis, noncleaved FAD domain and protease were separated from the FAD domain without a His_6_ tag by nickel affinity chromatography. The flowthrough, containing processed NqrF-FAD domain, was dialyzed overnight against 50 mM HEPES-NaOH (pH 7.0), 5% glycerol (vol/vol), and 0.1 mM EDTA, concentrated to 15 mg/ml, and stored in liquid nitrogen.

### Isolation and solubilization of *P. bryantii* membranes under oxic conditions.

Cells were harvested at an OD_600_ of 2.0 by centrifugation at 9,000 × *g* for 30 min (4°C). The cells were washed twice in 20 mM Tris-H_2_SO_4_ (pH 7.5), 50 mM K_2_SO_4_. Cell disruption was performed as described earlier (48), with some modifications. Cells (10 g wet weight) were resuspended in 30 ml 20 mM Tris-H_2_SO_4_ (pH 7.5) containing 50 mM K_2_SO_4_, 5 mM MgSO_4_, 1 mM dithiothreitol, 1 mM phenylmethylsulfonyl fluoride, 0.1 mM diisopropyl fluorophosphate, and traces of DNase I (Roche). The suspension was passed three times through an Emulsiflex C3 high-pressure homogenizer (Avestin) at 20,000 lb/in^2^. Cell debris and unbroken cells were removed by centrifugation at 27,000 × *g* for 30 min at 4°C. Membranes were collected by ultracentrifugation at 50,000 rpm (Beckman Ti70 rotor) for 1 h at 4°C, washed once in 20 mM Tris-H_2_SO_4_ (pH 7.5) containing 50 mM K_2_SO_4_ and 5% (vol/vol) glycerol, and resuspended in the same buffer. The membrane suspension (10 mg protein/ml) was frozen by pipetting aliquots of 30 μl into a reservoir of liquid N_2_. The frozen droplets were collected and stored in liquid N_2_ until further use.

For the solubilization of the membranes, different detergents were tested. 1 ml of membranes (10 mg) was incubated with 4% digitonin (wt/vol), 5% Triton X-100 (vol/vol), or 2.5% DDM (wt/vol) in a total volume of 1.5 ml. Protein/detergent ratios were 1:6 for digitonin, 1:7.5 for Triton X-100, and 1:3.75 for DDM. After incubation of membranes with the detergent solutions for 2 h at 6°C, the membrane suspensions were ultracentrifuged at 50,000 rpm (Beckman Ti70 rotor) for 30 min at 4°C. Supernatants containing solubilized membrane proteins were frozen and stored in liquid N_2_ as described above for membrane suspensions.

### Isolation and solubilization of *P. bryantii* membranes under anoxic conditions.

Anoxic cells of *P. bryantii* (10 g wet weight in 30 ml cell lysis buffer) were broken by the Emulsiflex C3 high-pressure homogenizer, which was operated under exclusion of O_2_ (49). For anoxic membrane isolation and solubilization, all steps described for oxic preparation were performed in an anaerobic chamber (Coy Laboratory Products) under an atmosphere of 5% H_2_ and 95% N_2_. O_2_ levels were monitored continuously (CAM-12; Coy Laboratory Products). All plastic materials were placed in the glove box several weeks before use. Buffers and other substances were made anoxic by flushing with N_2_ before entering the chamber. Outside the anaerobic chamber, samples were handled in gas-tight vials.

### Size exclusion chromatography.

DDM solubilizate (2 ml, 20 mg) was loaded on a Cytiva HiLoad 16/600 Superdex 200-pg column connected to a chromatographic system (Äkta Explorer). The absorbance at 400 nm was recorded. Elution was performed with 20 mM Tris-H_2_SO_4_ (pH 8.0), 50 mM K_2_SO_4_, 5% glycerol, and 0.03% (by weight) DDM. Fractions of 1 ml were collected, concentrated by ultrafiltration (membrane cutoff, 100 kDa) and analyzed by SDS-PAGE, immunostaining, and Vis spectroscopy.

### Quinone isolation and identification.

Quinone extraction from *P. bryantii* cells was performed as described in reference 50. The extracted quinones were separated by using reverse-phase HPLC (RP-HPLC) and an OmniSpher 5 C_18_ 150- by 4.6-mm column from Agilent. The Hitachi LaChrom Elite HPLC system was equilibrated with a 7:3 mixture of methanol-isopropanol with a flow rate of 1 ml min^−1^. After equilibration, 100 μl of the quinone extract was injected into the HPLC, and elution was followed for 80 min. The fractions absorbing at 260 nm were collected, and the solvent was evaporated. The precipitated quinones were dissolved in 50 μl chloroform, and high-resolution mass spectrometry (HR-MS) was performed on a Finnigan LCQ Deca LC/MS system (Thermo Scientific).

### Enzyme kinetics.

If not indicated otherwise, enzyme kinetics were carried out in a 1-cm quartz cuvette at 20°C using a diode array spectrophotometer (Black-comet; StellarNet Inc.). The detector and the light source (SL5 UV-Vis lamp; StellarNet Inc.) were placed outside the anaerobic chamber, whereas the cuvette holder was placed inside. These three components were connected with fiber optic cables. NADH oxidation was followed at 340 nm (ε_NADH_ = 6.22 mM^−1 ^cm^−1^) (51) in buffer (20 mM potassium phosphate [pH 7.5], 200 mM NaCl) containing 100 μM DMN (ε_DMN_ = 15.2 mM^−1 ^cm^−1^). DMN reduction was monitored simultaneously at 270 to 290 nm (52). The stimulation of NADH oxidation and quinone reduction activities of DDM solubilizates by Na^+^ was determined as described previously for *P. bryantii* membranes (7). The residual Na^+^ concentration of the reaction buffer (20 mM Tris H_2_SO_4_ [pH 7.5]) was 10 μM. Fumarate reduction assays were performed in buffer (20 mM potassium phosphate [pH 7.5]) containing benzyl viologen (∼0.5 mM), which was reduced by adding sodium dithionite crystals to achieve an absorbance of 1 at 564 nm (53). Then, 50 to 100 μg of protein was added, followed by the addition of 10 mM fumarate. Decrease in absorbance of benzyl viologen was monitored at 564 nm (ε_BV_ = 19.5 mM^−1 ^cm^−1^). The fumarate reduction assay was performed in the anaerobic chamber using anoxic materials and buffers. Linear regression analysis of initial data points was performed to calculate the initial rates (*n* = 3).

### UV/Vis difference spectra.

The method described in reference 54 was modified as follows. The absorption spectrum of reduced cofactors in membranes and solubilized membranes was compared with an aliquot of the same sample with cofactors in their oxidized state using a double beam UV/Vis spectrophotometer (Perkin Elmer; Lambda 16) at 20°C. Light was split by a half mirror, passing separately through the reference sample (beam 1) and through the test sample (beam 2). The light intensities passing through the sample and through the reference were compared. The range of 300 to 800 nm was monitored with an interval of 1 nm and a scan speed of 480 nm/min. The software Lambda-SPX calculated the difference in absorbance of beam 2 and that of beam 1 at a given wavelength. Cellular fractions, buffers, and reactants were anoxic or were made anoxic by flushing with N_2_ and were added to the cuvettes in the anaerobic chamber. The filled cuvettes were sealed gas tight with rubber stoppers to be placed into the photometer outside, where the recordings were started immediately (4 min after addition of reactants to the anaerobic chamber). Membranes and solubilized membranes (with 5% Triton X-100) were analyzed at a concentration of ∼500 μg of protein per ml in 20 mM potassium phosphate buffer, pH 7.5. To the cuvette placed in beam 2, reactants were added, as specified below. To obtain baseline spectra, which reflected the turbidity of the sample for subsequent correction, aliquots of air-oxidized membranes, or air-oxidized solubilized membranes, were placed in beam 1 and beam 2.

The first set of experiments aimed the identification of all redox cofactors with putative Vis absorbances in membranes of *P. bryantii*. To this end, air-oxidized membranes were analyzed in beam 1. In beam 2, an aliquot of this membrane suspension mixed with crystals of solid sodium dithionite, to fully reduce the cofactors, was analyzed. The difference spectrum of dithionite-reduced minus air-oxidized membranes was recorded.

In the second set of experiments, we addressed the putative electron transfer between the NQR and the QFR in membranes and solubilized membranes of *P. bryantii*. As a reference, air-oxidized membranes or solubilized membranes in 20 mM potassium phosphate buffer (pH 7.5) supplemented with 200 mM Na_2_SO_4_ and 175 μM DMN were placed in beam 1. In beam 2, an anoxic aliquot treated with 125 μM NADH was analyzed. The difference spectrum of NADH-reduced minus air-oxidized membranes or solubilized membranes was recorded.

In the third set of experiments, the reoxidation of NADH-reduced solubilized membranes with fumarate was investigated. Here, we used two aliquots containing anoxic NADH-reduced membranes or solubilized membranes. To one aliquot, 12.5 mM fumarate was added in the anaerobic chamber, and the cuvette was placed in beam 2. The difference spectrum of fumarate-reoxidized (beam 2) minus NADH-reduced membranes, or solubilized membranes (beam 1), was recorded.

In the fourth set of experiments, succinate was used as the reducing agent. In beam 1, we placed a cuvette with an aliquot of air-oxidized, solubilized membranes. In beam 2, an aliquot of anoxic, solubilized membranes was allowed to react with 12.5 mM succinate disodium salt. The difference spectrum of succinate-reduced minus air-oxidized solubilized membranes was recorded.

All experiments were repeated at least three times. Representative difference spectra are presented.

### Analytical methods.

Protein concentration was determined spectrophotometrically with the bicinchoninic acid (BCA) method (55) using the reagent from Pierce. Bovine serum albumin served as the standard. Sodium was determined by flame atomic absorption spectroscopy (AA240 instrument; Agilent Technologies). d-Glucose and acetate concentrations were determined as described previously (20). Gas chromatography–time-of-flight (GC-TOF) mass spectrometry was used to identify metabolites in the supernatant of a 1-liter *P. bryantii* overnight culture (OD_600_, ∼2) (56). Succinate was measured with an ion chromatograph (761 Compact IC; Metrohm) equipped with a Metrosep organic acid column (particle size, 9 μm; 250 by 7.8 mm; Metrohm) and an electron conductivity detector. To 9 ml 0.5 mM H_2_SO_4_, 1 ml of culture supernatant was added, and an aliquot of 20 μl was analyzed under isocratic conditions in 0.5 mM H_2_SO_4_ at a flow rate of 0.5 ml min^−1^.

### Mass spectrometry.

For the identification of proteins separated on BN or SDS gels, protein bands of interest were cut out and subjected to tryptic digestion (57). Nano-LC electrospray ionization (ESI) MS/MS experiments were performed on an Ultimate 3000 RSLCnano system (Dionex, Thermo Fisher Scientific, Germany) coupled to a Q-Exactive HF-X mass spectrometer (Thermo Fisher Scientific, Germany) using a NanosprayFlex source (Thermo Fisher Scientific, Germany). Tryptic peptides were directly injected onto a precolumn (μ-precolumn C_18_ PepMap100; 300 μm, 100 Å, 5 μm by 5 mm; Thermo Fisher Scientific) and an analytical column (NanoEase M/Z HSS C_18_ T3; 1.8 μm, 100 Å, 75 μm by 250 mm; Waters GmbH, Germany) operated at constant temperature of 35°C.

Gradient elution was performed at a flow rate of 300 nl/min using a 30-min gradient from 2% to 55% solvent B (0.1% formic acid, 80% acetonitrile) in solvent A (0.1% formic acid). The Q-Exactive HF-X instrument was operated under the control of Xcalibur software (version 4.3; Thermo Fisher Scientific, Inc., USA). Internal calibration was performed using lock-mass ions from ambient air (58). Survey spectra (*m/z* = 300 to 1,800) were detected in the Orbitrap instrument at a resolution of 60,000 at *m/z* 200. Data-dependent MS/MS mass spectra were generated for the 20 most abundant peptide precursors in the Orbitrap using high-energy collision dissociation (HCD) fragmentation at a resolution of 15,000 with normalized collision energy of 27.

Mascot 2.6 (Matrix Science, UK) was used as the search engine for protein identification. Spectra were searched against the protein databases of *Prevotellaceae* using FASTA-formatted protein sequences retrieved from NCBI (https://www.ncbi.nlm.nih.gov/protein/?term=Prevotellaceae) (June 2018). Search parameters specified trypsin, allowing three missed cleavages, a 5-ppm mass tolerance for peptide precursors and 0.02-Da tolerance for fragment ions. Methionine oxidation was allowed as a variable modification, and carbamidomethylation of cysteine residues was set as a fixed modification. The Mascot results were transferred to Scaffold software 4.8.6 (Proteome Software, USA). Peptide identifications were accepted with a peptide probability greater than 80.0% as specified by the Peptide Prophet algorithm (59). Proteins had to be identified by at least two peptides and a protein probability of at least 99% to be accepted. Protein probabilities were assigned by the Protein Prophet algorithm (60).

### NMR spectroscopy.

To detect succinate and fumarate in enzymatic assays by 1D ^1^H, 2D ^1^H, and ^13^C NMR, NADH oxidation by *P. bryantii* membranes was monitored spectroscopically under anoxic conditions as described above. Enzymatic activity was completed when no decrease of absorbance was observed, and subsequently, the sample was dried at room temperature with a vacuum concentrator (Eppendorf concentrator; program V-AQ). Afterward, the pellet was resuspended in 50 mM Na_2_HPO_4_ (pH 7) and 5 mM 3-trimethylsilyl propionic-2,2,3,3 acid sodium salt (TSP; Sigma-Aldrich) as an internal reference for ^1^H and ^13^C chemical shift calibration. Note that the phosphate buffer contained D_2_O instead of H_2_O. The resuspended sample was used to fill NMR tubes for 1D ^1^H, 2D ^1^H, and ^13^C measurements. NMR spectra were recorded using a Bruker Avance III HD NMR 600 MHz spectrometer equipped with a 5-mm BBO Prodigy cryoprobe. For structural identification of fumaric and succinic acid, 1D ^1^H as well as 2D heteronuclear NMR experiments (gradient-selected heteronuclear single quantum coherence [*g*HSQC] and gradient-selected heteronuclear multiple-bond correlation [*g*HMBC]) (61) were recorded at 298 K. For acquisition, processing and evaluation of NMR spectra, the software TopSpin 3.5pl7 (Bruker) was used. To monitor NADH oxidation and succinate formation by DDM solubilizates over time, NADH oxidation was followed anoxically at 340 nm in 20 mM potassium phosphate (pH 7.5), 100 mM Na_2_SO_4_, containing 10 mM NADH, 20 mM DMN, 10 mM fumarate, and 80 μg DDM-solubilized membranes of *P. bryantii*. Three assay mixtures were prepared, and the reactions were stopped after 1, 2, and 5 min by rapid freezing of the mixture in liquid N_2_. Note that NADH oxidation was completed after 5 min. The same experiment was conducted with 3 μM AgNO_3_ added to the assay. A 4-fold volume of acetone was added to the reaction mixtures. After storage overnight at −20°C, precipitated proteins were sedimented by centrifugation (5 min at 14,500 × *g*), and supernatants were analyzed by NMR spectroscopy.

### 1D PAGE and activity staining.

Denaturing polyacrylamide gel electrophoresis (SDS-PAGE) was performed with a 12% polyacrylamide gel. Protein and membrane suspensions were diluted in 5× SDS sample buffer (500 mM dithiothreitol [DTT], 1 M Tris-HCl [pH 6.8], 5% SDS, 28.8% glycerol [wt/vol], bromophenol blue) and boiled for 5 min before loading on SDS gel. For the blue native (BN) PAGE, ServaGel native gels (Serva) with a gradient from 4% to 16% acrylamide were used. The electrophoresis was performed following the instructions of the manufacturer, modified as follows. First, the gel was run at 90 V for 30 min. Then the voltage was increased to 100 V for ∼1 h. When 1/3 to 1/2 of the electrophoresis was completed, 1× anode buffer (50 mM bis-Tris [pH 7.0]) with 0.002% (wt/vol) Coomassie blue was changed to 1× anode buffer. The cathode buffer was always 50 mM Tricine, 15 mM bis-Tris (pH 7.0). The voltage was further increased to 200 V until the blue front reached the bottom of the gel. Note that the Coomassie dye was added to the anode buffer and not to the cathode buffer to improve detection of bands after activity staining.

To detect NADH-oxidizing proteins in gels, nitroblue tetrazolium (NBT) staining was performed as described in reference 62. The purified His-tagged NQR complex (48) served as the positive control. To detect fumarate reductase, the gel was placed in the anaerobic chamber and incubated with 10 ml 50 mM potassium phosphate (pH 7.5), 10 mM benzyl viologen, and 2 mg of solid sodium dithionite. The gel was shaken gently for 10 min. Sodium fumarate (2 mM) was added, and the gel was incubated for another 5 min. Fumarate reductase activity resulted in a clear band, due to oxidation of benzyl viologen with fumarate as the electron acceptor. For documentation, the gel was transferred to another tray filled with 50 mM potassium phosphate (pH 7.5) and photographed.

### 2D PAGE.

The stability of the NQR/QFR supercomplex was studied with the help of 2D BN/SDS. For 2D SDS/BN-PAGE, 1D BN-PAGE was performed as described above. Afterward, the lane of interest was excised and incubated with 1% SDS and 1% β-mercaptoethanol for 2 h. Meanwhile, a 12% SDS separating gel was poured between two glass plates (approximately two-thirds high) with a 1.5-mm spacer. After polymerization, the 1D lane was excised and placed on top of the separating gel. To melt the lane to the 2D SDS gel, an SDS stacking gel was added. SDS-PAGE was performed as described above.

### In-gel fluorography.

Fluorescence of covalently bound flavins in NqrB, NqrC, and FrdA was detected using the ImageQuant LAS 4000 imager (λ_excitation_ = 460 nm; emission filter, Y515 CyTM2). As a positive control, the purified NqrC′ subunit was used. This protein is a truncated variant of the NqrC subunit of the V. cholerae NQR comprising the covalently attached FMN but lacking the N-terminal transmembrane helix (32). The molecular mass of NqrC′ is 25.38 kDa.

### Western blotting and immune detection of proteins separated by SDS-PAGE.

SDS-PAGE and Western blot analysis were performed as described previously (63). The nitrocellulose membrane was incubated for 1 h with polyclonal rabbit antiserum (1:20,000 dilution; BioGenes GmbH) containing antibodies against the NqrF-FAD domain of *P. bryantii.* The antiserum exhibited no cross-reactivity with purified NqrF-FAD domain of V. cholerae. After two washing steps with phosphate-buffered saline-Tween (PBST; 137 mM NaCl, 2.7 mM KCl, 1.8 mM KH_2_PO_4_, 10 mM Na_2_HPO_4_, and 0.05% Tween-20 [wt/vol]), the membrane was incubated for 1 h with horseradish peroxidase (HRP)-conjugated secondary anti-rabbit immunoglobulin antibodies (1:3,000 dilution). Immune detection was performed using the chemiluminescence Clarity Western enhanced chemiluminescence (ECL) blotting substrates (Bio-Rad). Exposure (<1 min) and detection were performed with Image Quant LAS 400 (GE Healthcare). The purified NqrF-FAD domain of *P. bryantii* was used as a positive control.

### Quantitative real-time PCR analysis.

Total RNA from *P. bryantii* was isolated from 2 ml of cultures in mid-exponential phase. Cells were harvested by centrifugation at 15,000 rpm for 1 min. Cells were resuspended in 600 μl RLT Plus buffer provided with the RNeasy Plus minikit (Qiagen). Cell lysis and RNA isolation were performed with the RNeasy PowerMicrobiome kit (Qiagen). Since the RNA preparation was still contaminated with genomic DNA, a second DNase I degradation step was performed using the RNA Clean & Concentrator kit from Zymo Research. RNA was quantified with the help of the NanoDrop 2000c spectrophotometer (Thermo Scientific). The quality of the RNA was confirmed by gel electrophoresis (Fig. S6) and PCR.

cDNA synthesis was performed as recommended by the standard protocol of the First Strand cDNA synthesis kit (Thermo Scientific), using random hexamer primers. The amount of total RNA used to synthesize cDNA was 1 μg. The thermal cycling parameters for the RT reactions were 25°C for 5 min, 37°C for 60 min, and finally 70°C for 5 min to inactivate the reverse transcriptase. Afterward, a PCR was done with cDNA as the template and gene-specific primers. Primers were designed using NCBI sequence sources and the Primer3 online primer designing tool (64). The corresponding PCR products were confirmed by sequencing (Eurofins). Primers were designed for *nqrF* D8DWB6_PREBR (*nqrF* forward primer, 5′CTCAGGTCGGTTTCCAGGAT3′, and *nqrF* reverse primer, 5′ATTTGGATTGGTGGTGGTGC3′), *frdA* D8DXM6_PREBR (*frdA* forward primer, 5′CAGGGTGGTATCAATGCTGC3′, and *frdA* reverse primer, 5′GTTCATTCGGTGGTGCTCAG3′), *rnfG* A0A1H9FEX3_PREBR (*rnfG* forward primer, 5′CAGAAAAGACCCTTGCTGCA3′, and *rnfG* reverse primer, 5′CGGTGCAGCAGTAGAAAGTG3′) and for the reference gene *recA* D8DYF7_PREBR (*recA* forward primer, 5′CTTTCGACCGCTTCTATGCC3′, and reverse primer, 5′AGGCGATATGGGTGACAACA3′). In this PCR, reverse transcriptase minus controls were conducted for each biological replicate to assess for genomic DNA contamination of the RNA sample and a no-template negative control (NTC) was performed to guarantee the purity of the reagents.

RT-qPCRs were performed with the Platinum SYBR green qPCR SuperMix-UDG kit from Invitrogen. For temperature cycling and fluorescence measurements a Bio-Rad CFX 96 cycler and the appropriate CFX Manager software (Bio-Rad) were used. Samples from two biological replicates were measured in three technical replicates each. Mean values and standard deviations of these data were used to compare expression by the 2^−ΔΔ^*^CT^* method (where *C_T_* is the threshold cycle) (65).
