# Supplementary material for: A Sodium-Translocating Module Linking Succinate Production to Formation of Membrane Potential in Prevotella bryantii
Source: Appl Environ Microbiol. 2021 Oct 14;87(21):e01211-21. doi: 10.1128/AEM.01211-21 (PMC8516057; doi:10.1128/AEM.01211-21)
Supplement: Supplemental file 1 — Figures S1 to S7, Tables S1 to S3 and S6. Download AEM.01211-21-s0001.pdf, PDF file, 1.7 MB [file aem.01211-21-s0001.pdf]

Supplementary Information for

A sodium-translocating module linking succinate production to formation of a membrane potential in *Prevotella bryantii*

Lena Schleicher*†, Andrej Trautmann*‡, Dennis Stegmann*†, Günter Fritz*†, Jochem Gätgens¶, Michael Bott¶, Sascha Hein§, Jörg Simon§, Jana Seifert*‡, Julia Steuber*†#

* HoLMiR- Hohenheim Center for Livestock Microbiome Research, University of Hohenheim, Leonore-Blosser-Reisen-Weg 3, 70599 Stuttgart, Germany

† Institute of Biology, University of Hohenheim, Garbenstraße 30, 70599 Stuttgart, Germany

‡ Institute of Animal Science, University of Hohenheim, Emil-Wolff-Straße 8, 70599 Stuttgart, Germany

§ Microbial Energy Conversion and Biotechnology, Department of Biology, Technical University of Darmstadt, Schnittspahnstraße 10, 64287 Darmstadt, Germany

¶ Institute of Bio- and Geosciences, IBG-1: Biotechnology, Forschungszentrum Jülich, 52425 Jülich, Germany

# Correspondence: Julia Steuber, julia.steuber@uni-hohenheim.de, +49 711 459 22228

This PDF file includes:

Figures S1-S7

Tables S1, S2, S3, S6

References

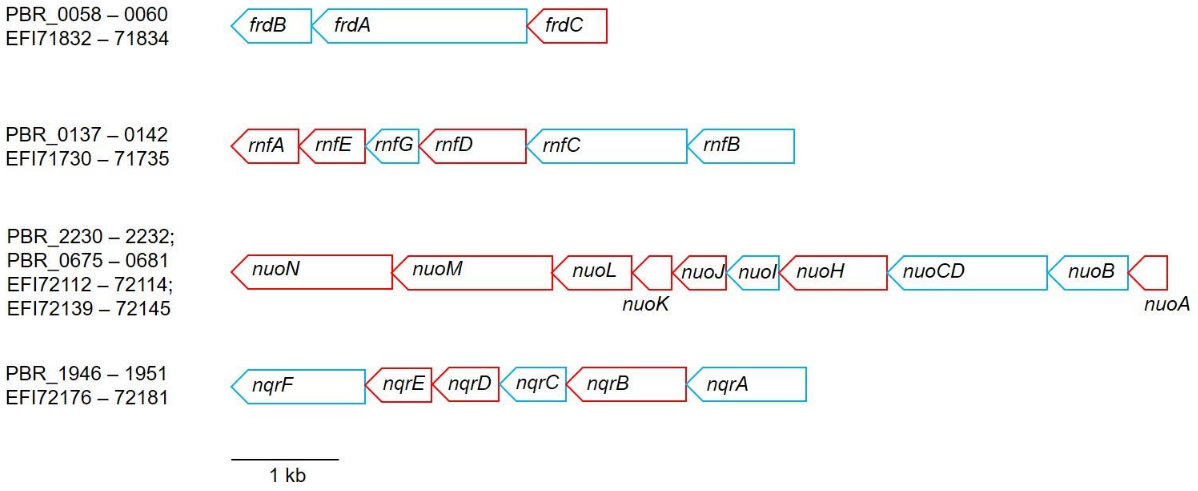

Figure S1: Predicted operons encoding respiratory complexes of *P. bryantii* b$_1$4 with corresponding operon reading frames (ORF). NCBI accession numbers with the prefix EFI are assigned to genes based on ORFs. Subunits of QFR are encoded by *frdC* (686 bp), *frdA* (1979 bp) and *frdB* (758 bp). Subunits of RNF are encoded by *rnfB* (917 bp), *rnfC* (1418 bp), *rnfD* (992 bp), *rnfG* (572 bp), *rnfE* (626 bp) and *rnfA* (602 bp). The 11-subunit complex related to the Nuo complex (NDH-I, or complex I) is assigned to a gene cluster comprising *nuoA* (350 bp), *nuoB* (764 bp), *nuoCD* (1574 bp), *nuoH* (1094 bp), *nuoI* (281 bp), *nuoJ* (521 bp), *nuoK* (308 bp), *nuoL* (860 bp), *nuoM* (1505 bp), and *nuoN* (1469 bp). Note that genes coding for homologs of NuoE, -F and -G subunits which represent the NADH-oxidizing module in complex I are absent in *P. bryantii*. Subunits of the NQR complex are encoded by *nqrA* (1349 bp), *nqrB* (1157 bp), *nqrC* (632 bp), *nqrD* (629 bp), *nqrE* (626 bp) and *nqrF* (1268 bp). Red, genes coding for membrane bound subunits; blue, genes coding for peripheral subunits.

NqrA

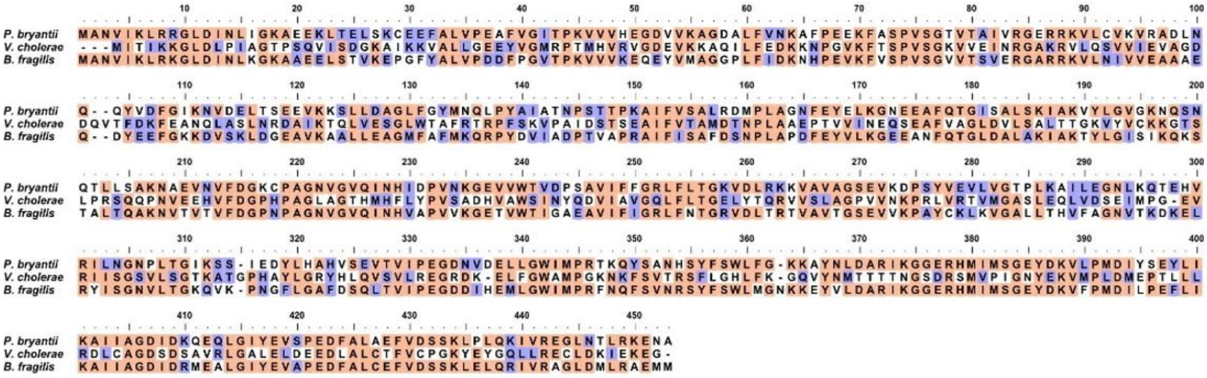

NqrB

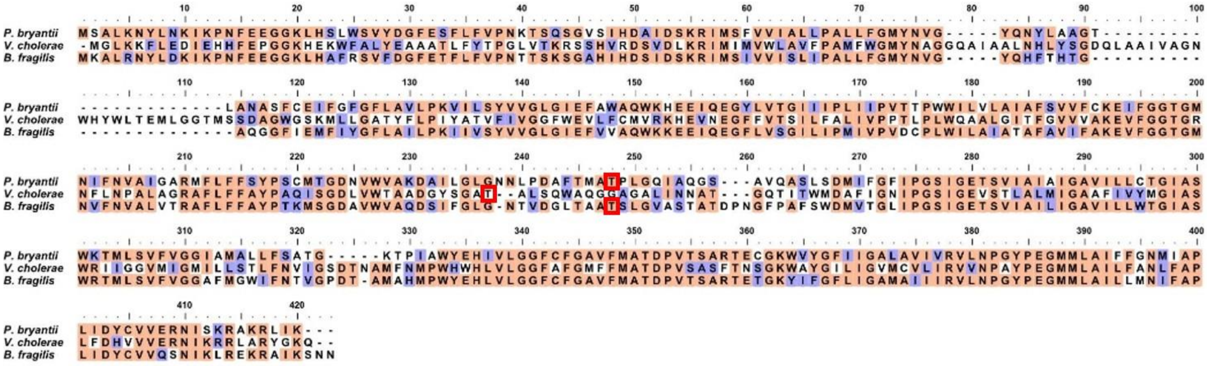

NqrC

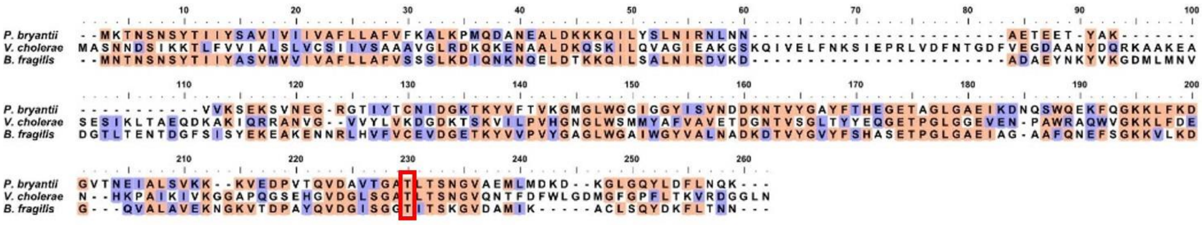

NqrD

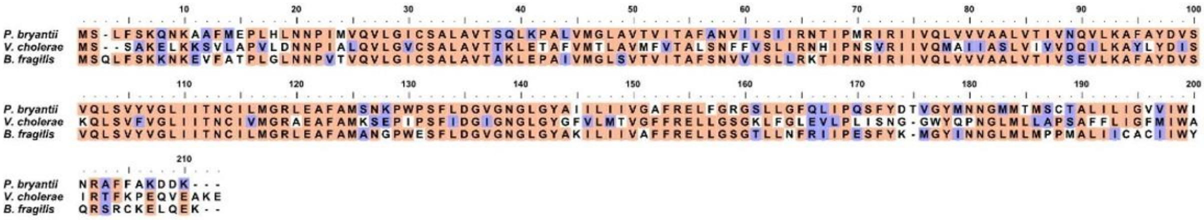

NqrE

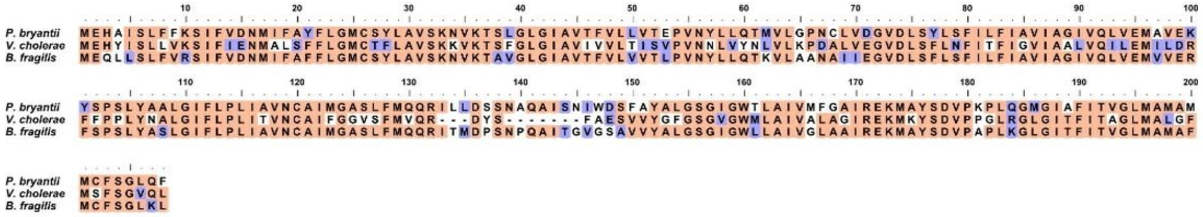

NqrF

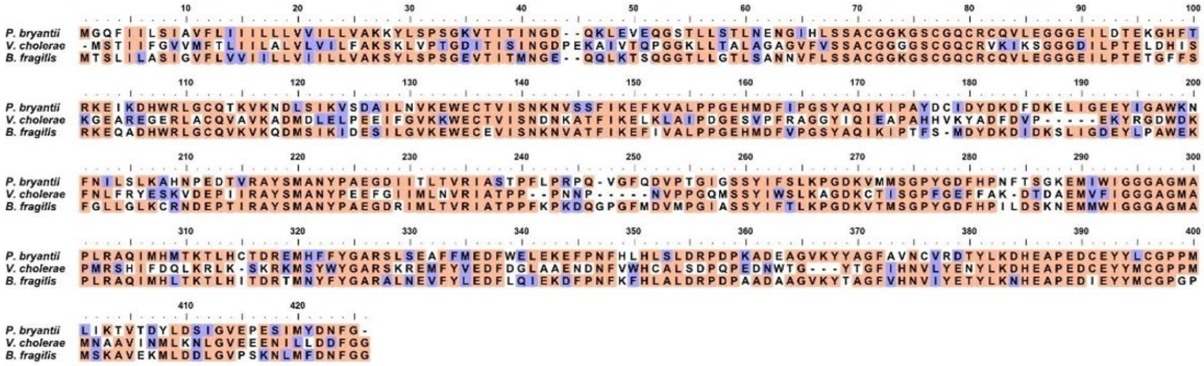

Fig. S2: Protein sequence alignments of the NQR subunits NqrA, NqrB, NqrC, NqrD, NqrE and NqrF from *Prevotella bryantii, Vibrio cholerae* and *Bacteroides fragilis*. Orange, identical amino acids; purple, similar amino acids. Conserved amino acids for flavin binding are highlighted in red.

FrdA

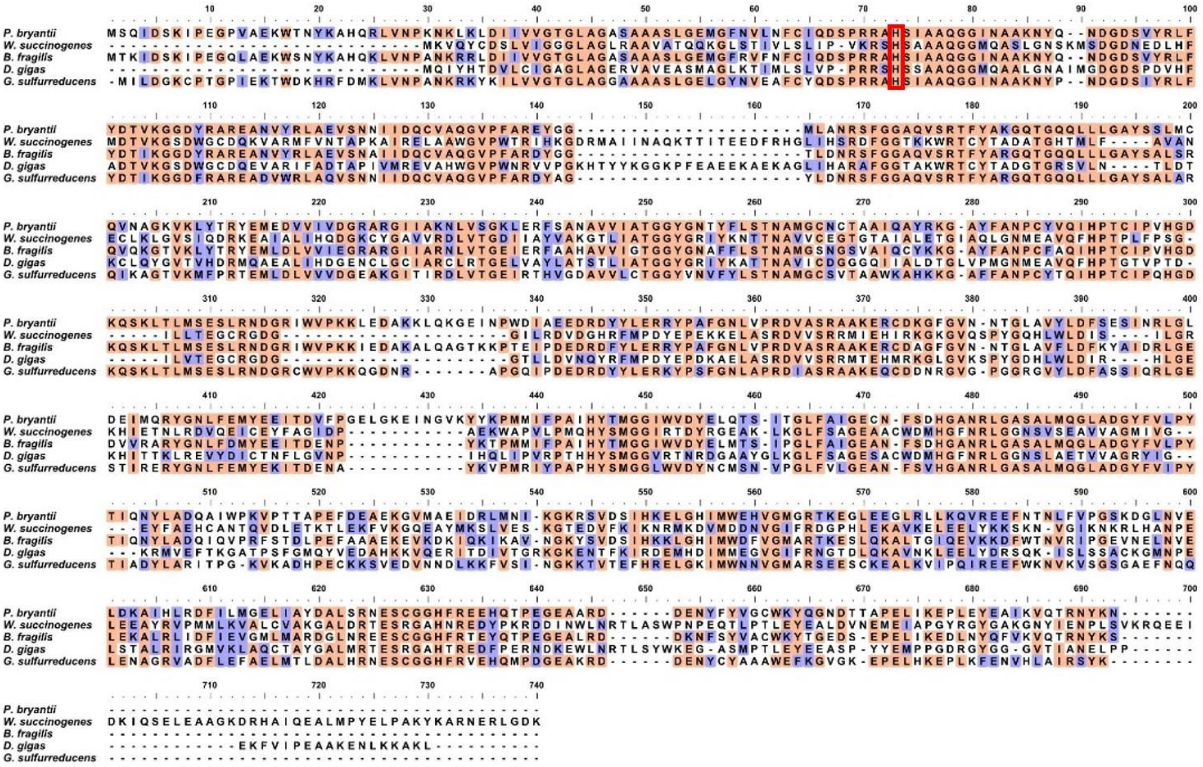

FrdB

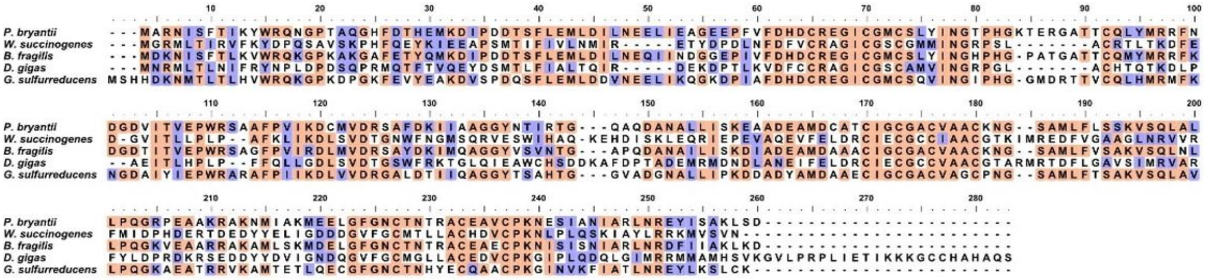

FrdC

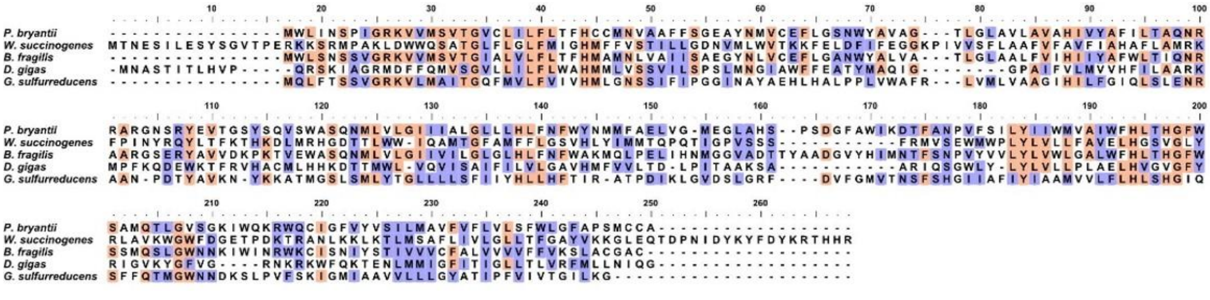

Fig. S3: Protein sequence alignments of the QFR subunits FrdA, FrdB and FrdC from *Prevotella bryantii, Wolinella succinogenes, Bacteroides fragilis*, *Desulfovibrio gigas* and *Geobacter sulfurreducens* are shown. Orange, identical amino acids; purple, similar amino acids. Conserved amino acids for flavin binding are highlighted in red.

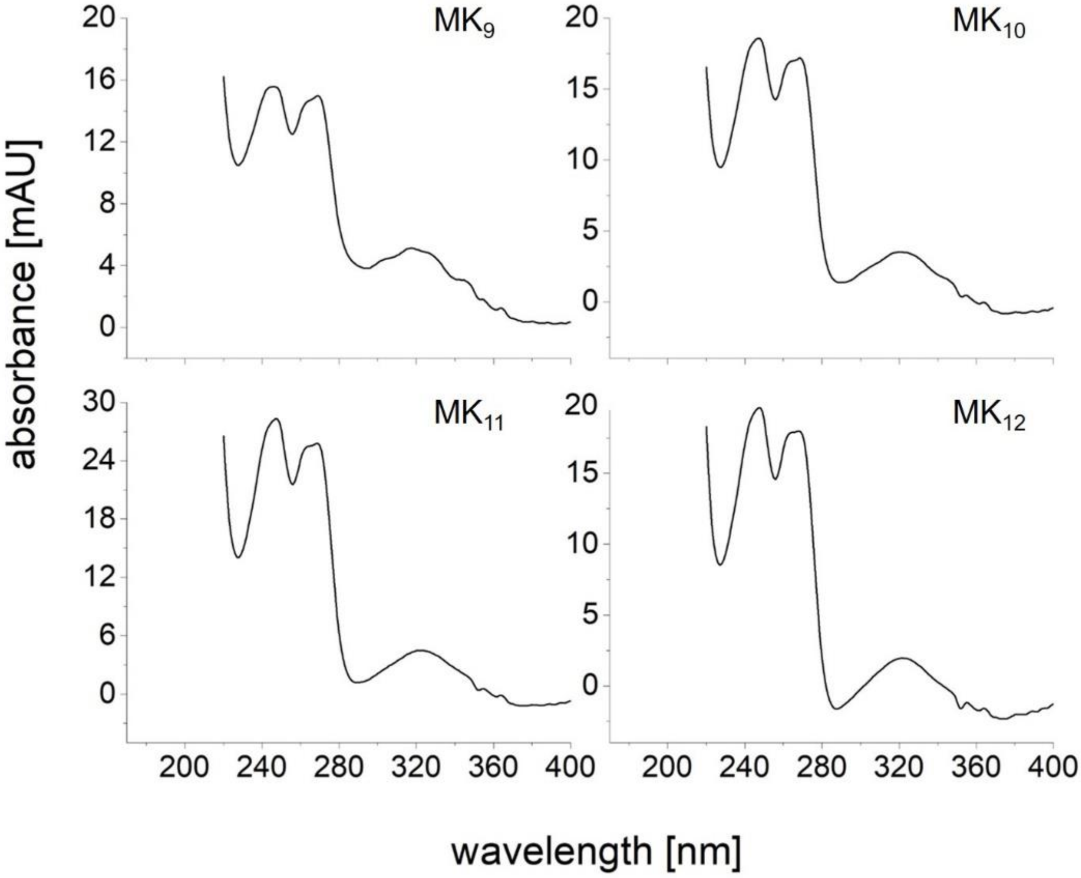

Figure S4: UV/VIS absorption spectra of menaquinones of *P. bryantii* membranes. Quinones were extracted with organic solvent and separated by HPLC. Fractions assigned to MK9, MK10, MK11 and MK12 by mass spectrometry revealed typical maxima at 246 nm, 264 nm and 329 nm (1).

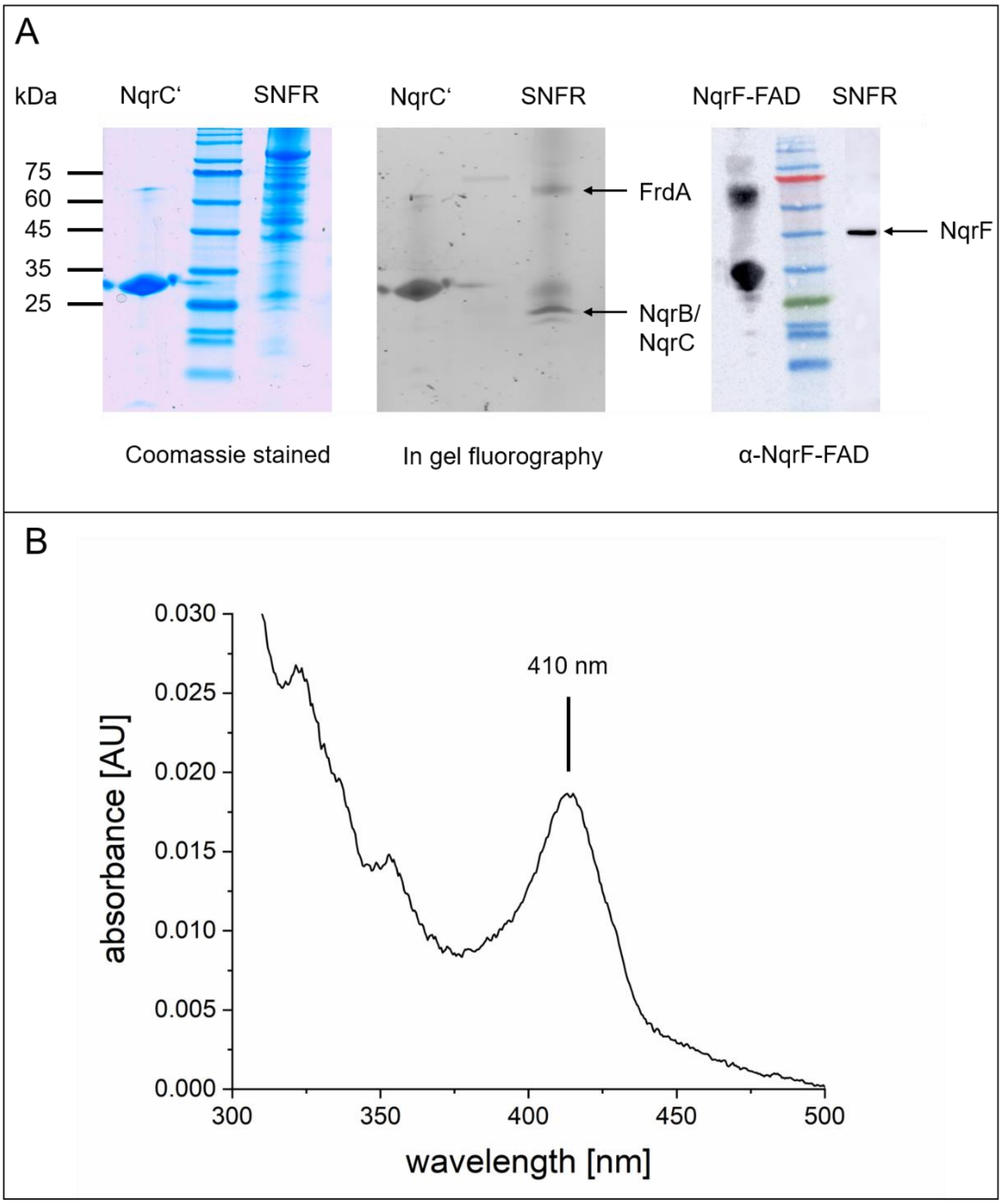

Figure S5: Characterization of the SNFR complex from size exclusion chromatography. A: Fractions eluting at 49 - 56 mL from the size exclusion column (fig. 4C) were concentrated, and 50 µg were separated by SDS PAGE. Left, after Coomassie staining; middle, fluorography for the detection of flavinylated proteins (with 2 µg flavinylated NqrC´ as positive control); right, Western Blot using anti-NqrF-FAD antibodies with purified NqrF-FAD domain (10 µg) as positive control. B: VIS spectrum of the SNFR (5 µg in 1 mL 20 mM Tris H_2SO_4 pH 8.0, 50 mM K_2SO_4, 5 % glycerol and 0.03 % DDM) with a maximum at 410 nm.

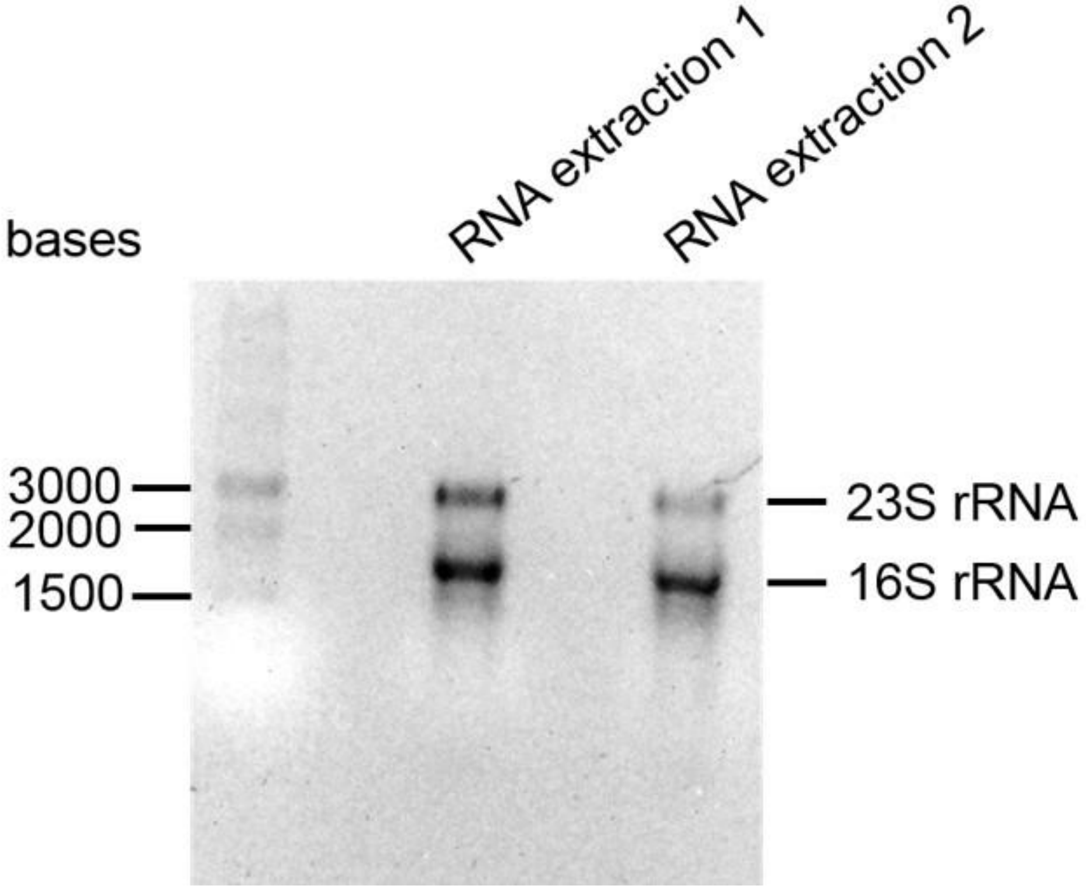

Figure S6: Total RNA extraction of *P. bryantii* from two biological replicates. Signals obtained from denaturing formaldehyde agarose gels electrophoresis represent the two characteristic ribosomal 23S and 16S rRNAs at 3000 bp and 1500 bp respectively. 2 µl of extracted RNA was mixed with an equal amount of 2x RNA Loading Dye (Thermo Scientific) and incubated for 10 min at 70 °C. Samples were loaded on a 0.75 % agarose TAE (40 mM Tris, 0.05 mM Na_2EDTA, 0.1 % acetic acid (v/v)) gel with 20 µl 37 % formaldehyde and 8 µl GelRed. The gel was run in TAE buffer for 1.5 h at 60 V.

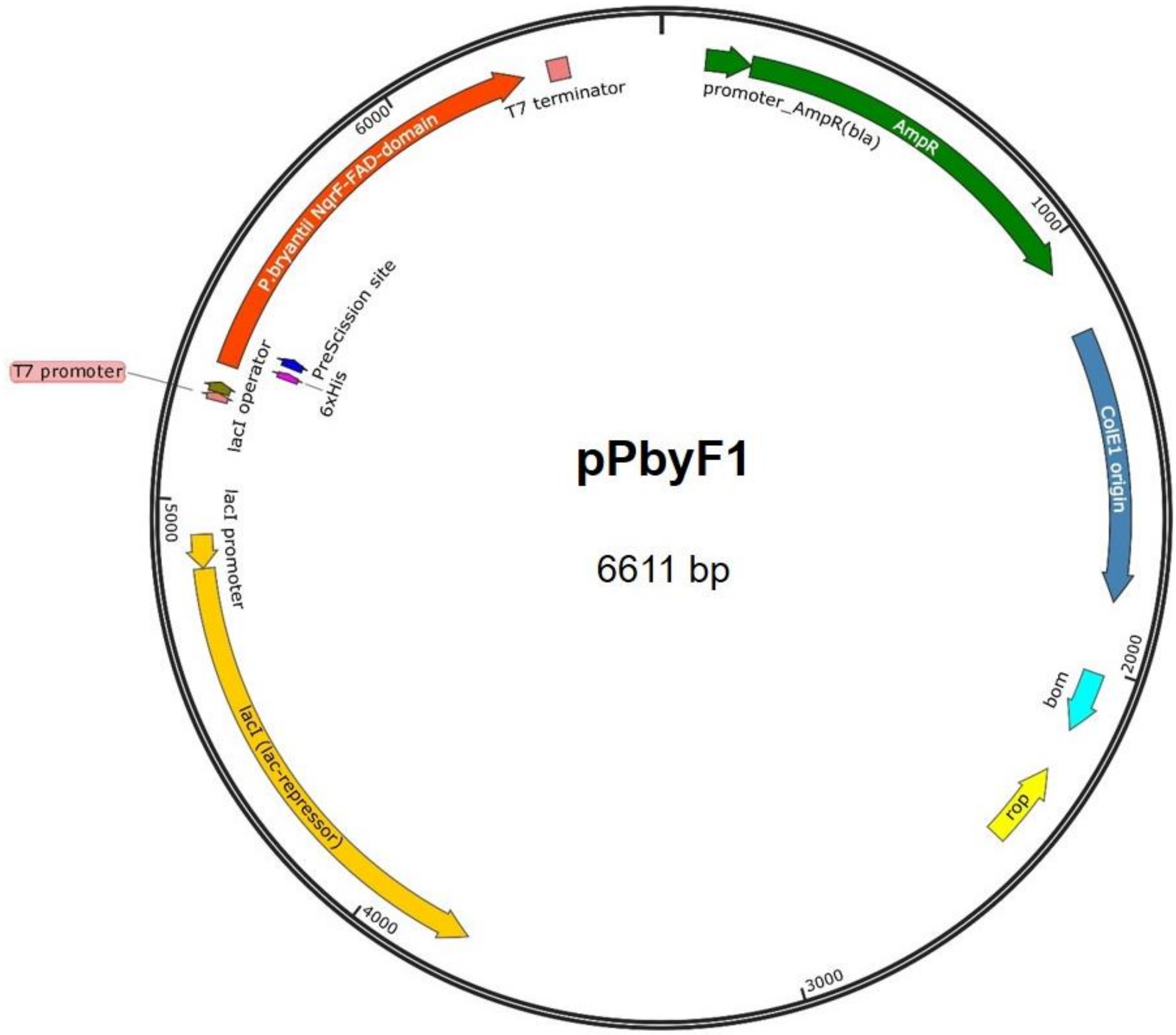

Figure S7: Schematic map of the expression vector for the *P. bryantii* NqrF-FAD domain. The ColE1 derived pET15b backbone (Novagen) contains genes for ampicillin resistance, for the lacI repressor protein, and a multiple cloning site (mcs) coupled to a T7-RNA-Polymerase promotor- and terminator-sequence. The codon optimized gene for the NqrF-FAD domain of *P. bryantii* comprising a His-tag and a protease (PreScission) site was inserted into the mcs.

Table S1: Overall hydrophobicity of subunits of NQR, RNF and QFR. Shown are the amino acid number, molecular mass and hydrophobicity of each subunit. The hydrophobicity was determined with the hydrophobicity index using GRAVY (2). This scale covers the range from - 2 to + 2. Proteins with a hydrophobicity > 0 are hydrophobic and proteins with a hydrophobicity < 0 are hydrophilic.

	**amino acid number**	**molecular mass [kDa]**	**hydrophobicity**
NQR			
NqrA	449	49.4	- 0.115
NqrB	385	41.7	+ 0.739
NqrC	210	23.2	- 0.297
NqrD	209	22.8	+ 0.912
NqrE	208	22.5	+ 1.023
NqrF	422	47.1	- 0.129
RNF			
RnfA	200	21.5	+ 1.217
RnfB	305	31.9	+ 0.114
RnfC	472	50.5	- 0.044
RnfD	330	35.3	+ 0.721
RnfE	194	20.5	+ 1.069
RnfG	190	19.7	+ 0.013
QFR			
FrdA	659	73.6	- 0.376
FrdB	252	27.6	- 0.239
FrdC	228	25.6	+ 0.864

Table S2: *In silico* tryptic digestion of subunits of NQR, RNF and QFR. Listed are the sequences of the predicted peptides and their expected masses. Only peptides > 500 Da are shown. Peptides identified by mass spectrometry are indicated in red (see also Table S3 and S6).

	**peptide mass [Da]**	**peptide sequences**
**NQR**		
NqrA	3721	SSIEDYLHAHVSEVTVIPEGDNVDELLGWIMPR
	2883	SLLDAGLFGYMNQLPYAIATNPSTTPK
(36 peptides)	2701	QEQLGIYEVSPEDFALAEFVDSSK
	1879	GEVVWTVDPSAVIFFGR
	1875	CPAGNVGVQINHIDPVNK
	1850	CEEFALVPEAFVGITPK
	1835	QYSANHSYFSWLFGK
	1584	VLPMDIYSEYLIK
	1552	GNEEAFQTGISALSK
	1527	DMPLAGNFEYELK
	1517	DPSYVEVLVGTPLK
	1511	ADLNQQYVDFGIK
	1405	FASPVSGTVTAIVR
	1263	NVDELTSEEVK
	1210	HMIMSGEYDK
	1204	NQSNQTLLSAK
	1140	ILNGNPLTGIK
	1092	NAEVNVFDGK
	1081	VVVHEGDVVK
	943	GLDINLIGK
	934	AGDALFVNK
	915	AIIAGDIDK
	876	AIFVSALR
	859	VAVAGSEVK
	857	AILEGNLK
	822	AYNLDAR
	802	EGLNTLR
	769	QTEHVR
	735	VYLGVGK
	720	AFPEEK
	690	LTELSK
	678	LFLTGK
	675	MANVIK
	598	LPLQK
	561	VLCVK
	502	VDLR
NqrB	6887	DAILGLGNNLPDAFTMATPLGQIAQGSAVQASLS<br>DMIFGFIPGSIGETSVIAIAIGAVILLCTGIASWKIMSFVV
(16 peptides)	5375	IALLPALLFGMYNVGYQNYLAAGTLANA<br>SFCEIFGFGFLAVLPK
	4521	HEEIQEGYLVTGIIIPLIIPVTTPWWILVLAIAFSVV

		FCK
	3473	VLNPGYPEGMMLAIFFGNMI APLIDYCVVER
	3257	TPIAWYEHIVLGGFCFGAVF MATDPVTSAR
	2385	LHSLWSVYDGFESFLFVPNK
	2343	MFLFFSYPSCMTGDNVWVAK
	2179	VILSYVVGLGIEFAWAQWK
	2114	TMLSVFVGGIAMALLFSATGK
	1867	EIFGGTGMNIFNVAIGAR
	1677	WVYGFIIGALAVIVR
	1545	TSQSGVSIHDAIDSK
	1119	IKPNFEEGGK
	651	NYLNK
	549	MSALK
	537	TECGK
NqrC (13 peptides)	3007	TNSNSYTIIYSAVIVIIVAFLLAFVFK
	2891	VEDPVTQVDAVTGATLTSNGVAEMLMDK
	2328	NTVYGAYFTHEGETAGLGAEIK
	1839	GMGLWGGIGGYISVNDDK
	1544	ALKPMQDANEALDK
	1497	NLNNAETEETYAK
	1396	GLGQYLDFLNQK
	1246	DGVTNEIALSVK
	1184	GTIYTCNIDGK
	1120	QILYSLNIR
	1034	DNQSWQEK
	756	YVFTVK
	661	SVNEGR
NqrD (9 peptides)	5772	AAFMEPLHLNNPIMVQVLGICSALAVTSQLKPALVMG LAVTVITAFANVIISIIR
	4767	GSLLGFQLIPQSFYDTVGYMNNGMMTMSCTALILIGV VIWINR
	3737	LEAFAMSNKPWPSFLDGVGNGLGYAIILIIVGAFR
	2845	AFAYDVSVQLSVYVGLIITNCILMGR
	2132	IIVQLVVVAALVTIVNQVLK
	731	NTIPMR
	712	MSLFSK
	621	ELFGR
	583	AFFAK
NqrE (6 peptides)	6822	TSLGLGIAVTFVLLVTEPVNYLLQTMVLGPNCLVDGVD LSYLSFILFIAVIAGIVQLVEMAVEK
	4142	ILLDSSNAQAISNIWDSFAYALGSGIGWTLAIVMFGAIR
	3757	MAYSDVPKPLQGMGIAFITVGLMAMAMMCFSGLQF
	3459	YSPSLYAALGIFLPLIAVNCAIMGASLFMQQR
	2619	SIFVDNMIFAYFLGMCSYLAVSK
	1223	MEHAISLFFK
NqrF (33 peptides)	3620	IASTPFLPRPQVGFQDVPTG IGSSYIFSLKPGDK
	2966	MGQFIILSIAVFLIIILLLV VILLVAK
	2757	LEVEQGSTLLSTLNENGIHL SSACGGK
	2465	TVTDYLDSIGVEPESIMYDN FG
	2323	DHEAPEDCEYYLCGPPMLIK
	2170	VALPPGEHMDFIPGSYAQIK

	2085	AYSMANYPAEGDIITLTVR
	2062	EFPNFHLHLSLDRPDPK
	2008	SLSEAFFMEDFWELEK
	1972	VMMSGPYGDFHPNFTSGK
	1591	CQVLEGGGEILDTEK
	1559	EMIWIGGGAGMAPLR
	1408	ELIGEEYIGAWK
	1316	IPAYDCIDYDK
	1263	YYAGFAVNCVR
	1209	EWECTVISNK
	1157	EMHFFYGAR
	1089	VTITINGDQK
	1038	AHNPEDTVR
	959	AQIMHMTK
	958	VSDAILNVK
	948	NFNILSLK
	845	TLHCTDR
	794	NVSSFIK
	751	YLSPSGK
	710	GSCGQCR
	689	NDLSIK
	689	ADEAGVK
	649	LGCQTK
	639	DTYLK
	617	GHFTR
	613	DHWR
	524	DFDK
**RNF**		
RnfA	6495	IDTALGMGAAVTFVMTLATIVTFLIQTYVLTPFHLQYLQ T LAFILVIAALVQMIEIILK
(7 peptides)	3589	MEYLLIFISAIFVNNIVLSQ FLGICPFLGVSK
	3327	TSPALYQALGVFLPLITTNC AVLGVAILVIQK
	3120	DYSLLQSVVYAFSTALGFAL ALILFAGIR
	2997	GMQGMSIVLVTAGLLALAFM GFSGLEGGLR
	817	EQQALTK
	664	TLFGLN
RnfB	3097	CPVGGDPVMGEVADLLGMAV ANTEPMVAVVR
	2828	MNFILIAVLVLGAIALVAAV ILYIVSK
(19 peptides)	2817	IAQVVEILPGANCGGCGFAG CGGLAEALVK
	2585	TCQAMNANGSGETGCGFGCL GCGDCTK
	2416	ACAFDAIHMNPETGLPEVDE EK
	2031	FEAITIENNLSYIDFNK
	1831	SVETVAQPAAASVNEETK
	1249	CVDECPTGAIIK
	1121	CNGTCTNRPR
	1045	GADAGSIEGIR
	1013	SCTAACIGCGK
	961	FAVQEDPR
	952	VYVQCVNK
	936	IAEYDGLR
	873	CTSCGACTK
	780	HIIELR
	703	VNFPVK

	514<br>504	AQPAK<br>VEEK
RnfC<br><br>(33 peptides)	4083<br>3166<br>2467<br>2400<br>2332<br>2325<br>2251<br>2192<br>2071<br>1612<br>1610<br>1573<br>1287<br>1192<br>1144<br>1079<br>1075<br>960<br>956<br>946<br>903<br>872<br>826<br>819<br>816<br>790<br>778<br>769<br>700<br>656<br>645<br>572<br>547	MFDQLEQEEVVSCISCGSCQFTCPAHRPLLDNIANGK<br>QVPAPPAIPVNVGAIVQNVGTAYAVYQAVMK<br>VGTLLAEAGGFVSAPVYSSVSGTVAK<br>KPAIIINVDEEDEWEESIDR<br>AEFVIINAVECEPYITSDYR<br>SDTLETLAAHPELTSEEIVNR<br>CVEACPMGLEPYLLATLSVNK<br>MGTPMSQLIEACGGLPDDDNK<br>LMMEHADEILVGVELLMK<br>QAIFMLSQHIGAPAK<br>VAGVTGMGGAGFPTFIK<br>GFIGIEENKPEAIR<br>IDNVYDATGYR<br>EHAGIEVVPLK<br>AVISLDVPVCK<br>IGGVHPEENK<br>GTNAITVLTGK<br>VLAGGPMMGK<br>QLVDAVVGR<br>NPSNFLVR<br>NKPLFER<br>ITAEIPTK<br>LCPPPTAK<br>LLTELCK<br>GAVMGIIR<br>EAQACIR<br>YPQGGEK<br>YTTVTGK<br>AKPADAK<br>VAELPK<br>LHTFK<br>AVVQR<br>AEAEK
RnfD<br><br>(12 peptides)	6402<br><br>4693<br><br>4311<br><br>3949<br><br>3945<br><br>3457<br>1848<br>1833<br>1831<br>947<br>554<br>551	IITWHIPVSILCTVFVFSGLMHMINPVYANPVYELLSGG<br>MLGAIFMATDYVTSPMTK<br>NMYGVIIALIPAVLVSLYYFGIGSAVVLLTSVAACVFFE<br>WAIAK<br>TKPQVLDGSAMLTGLLLGMNLPSNLPLWIIILGALIAIG<br>VGK<br>LPDTFTMLLGNPANGMGAGTIGEVCAAALLLGLIYML<br>VK<br>CFLLVSFPAQMTSWPTVGQLGSYLDAQTGATPLSVM<br>K<br>NWGSYPEGMSFAILIMNGFTPLINHYMKPK<br>MSFGGLGNNPFNPALVGR<br>GQLIYGVAIGFLTIVIR<br>LIVSLSPHAHGNDSVER<br>TGDASLLDR<br>YMLK<br>YGIAK

RnfE	5056	IPVFIVVIAAFVTILQMVMSAYAPDSINQALGLFIPLIVVN
		CIILGR
(8 peptides)	4795	ENPTFVLTLGMCPTLATTTSAINGFSMGLATMAVLICT
	4427	NFVISCIK
		ELLGAGSIFGINLLPETTNILLFILPPGAFITLGYLSAIINK
	2762	NSPLASIFDGIGIGLGFTGALTLLGCVR
	887	VLLNGMIK
	831	ITPDMVR
	755	AESFACK
	592	MSNIK
RnfG	3742	NVMGTNDLQVAEPVNVTETIDGKPVSFTIHATTDK
	3133	MVLVLVGVSLIIGGLLAYIN HLTEGPIAEK
(10	2923	VLVGFNPEGQILGYTILQHA ETPGLGAK
peptides)	1679	TLGAAVESVTGGFGGDLK
	1578	TGNAVDAITASTITSR
	1257	AINQAYAVYIK
	1153	NPADGDLHVSK
	851	AGDWFQK
	673	TLAAGIK
	601	GNIIGK
**QFR**		
FrdA	5257	YYKPMMIFPAIHYTMGGIWVDYELQTSITGLFAIGECN
		FSDHGANR
(51	3721	FSANAVVIATGGYGNTYFLSTNAMGCNCTAAIQAYR
peptides)	3628	LGASALMQGLADGYFVLPYTIQNYLADQAIWPK
	3607	LDIIVVGTGLAGASAAASLGEMGFNVLNFCIQDSPR
	2530	GAYFANPCYVQIHPTCIPVHGDK
	2451	YGNLFEMYEEITDVFPGELGK
	2367	GQTGQQLLLGAYSSLMCQVNAGK
	2273	GFGVNNTGLAVYLDFSESINR
	2244	LAEVSNNIIDQCVAQGVPFAR
	1827	DFILMGELIAYDALSR
	1652	ELGHIMWEHVGMGR
	1544	GEINPWDIAEEDR
	1539	DDENYFYVGCWK
	1482	EEFNTNLFVPGSK
	1450	YQGNDTTAPELIK
	1437	VPTTAPEFDEAEK
	1425	YEMEDVVIVDGR
	1354	EEHQTPEGEAAR
	1331	NYQNDGDSVYR
	1309	AHSIAAQGGINAAK
	1134	YPAFGNLVPR
	1092	EPLEYEAIK
	1075	LGLDEIMQR
	1050	LTLMSESLR
	1010	EYGGMLANR
	1006	NESCGGHFR
	1002	DGLNVELDK
	939	IPEGPVAEK
	908	SFGGAQVSR
	902	EGLEEGLR
	890	GVMAEIDR

	885	LFYDTVK
	858	DYYLER
	808	MSQIDSK
	785	SVDSIHK
	751	EANVYR
	711	WTNYK
	659	EINGVK
	642	IWVPK
	629	TFYAK
	618	LMNIK
	617	NLVSGK
	609	AIHLR
	575	LEDAK
	570	LVNPK
	567	GGDYR
	552	LYTR
	547	DVASR
	511	AHQR
	503	VQTR
	501	GIIAK
FrdB (20 peptides)	3967	DIPDDTSFLEMLDILNEELI EAGEEPFVFDHDCR
	2206	EAADEAMDCATCIGCGACVA ACK
	1880	EGICGMCSLYINGTPHGK
	1798	QNGPTAQGHFDTHEMK
	1678	VSQLALLPQGRPEAAK
	1548	FNDGDVITVEPWR
	1472	MEELGFGNCTNTR
	1430	TGQAQDANALLISK
	1155	NGSAMLFLSSK
	1149	IIAAGGYNTIR
	1143	GATTCQLYMR
	987	NESIANIAR
	832	SAAFPVIK
	822	NISFTIK
	820	ACEAVCPK
	738	DCMVDR
	710	EYISAK
	576	NMIAK
	567	SAFDK
	524	YWR
FrdC (6 peptides)	7550	VVMSVTGVCLILFLTFHCCMNVAAFFSGEAYNMVCEFLGSNWYAVAGTLGLAVLAVAHIVYAFILTAQNR
	6965	YEVTGSYSQVSWASQNMLVLGIIIALGLLLHLFNFWYNMMFAELVGMEGLAHSPSDGFAWIK
	4484	DTFANPVFSILYIIWMVAIWFHLTHGFWSAMQTLGVSGK
	3848	WQCIGFVYVSILMAVFVFLVLSFWLGFAPSMCCA
	1187	MWLINSPIGR
	574	IWQK

Table S3: Subunits of the NQR, QFR and RNF identified from *P. bryantii* membranes solubilized with 2.5 % DDM or 5 % Triton X-100 identified by mass spectrometry. Bands were excised from Coomassie stained 1D BN PAGE as indicated in fig. 2C. Identified subunit, total number of identified peptides, and peptide sequences are presented.

**Box number**	**Identified protein**	**Peptide number**	**Peptide sequences**
**1**	FrdA	29	SQIDSKIPEGPVAEK; IPEGPVAEK; IPEGPVAEKWTNYK; RAHSIAAQGGINAAK; AHSIAAQGGINAAK; NYQNDGDSVYR; LFYDTVK; LAEVSNNIIDQCVAQGVPFAR; EYGGMLANR; SFGGAQVSR; YEMEDVVIVDGR; NLVSGKLER; LTLMSESLR; GEINPWDIAEEDR; RYPAFGNLVPR; YPAFGNLVPR; LGLDEIMOR; VPTTAPEFDEAEK; VPTTAPEFDEAEKGVMAEIDR; ELGHIMWEHVGMGR; TKEGLEEGLR; EGLEEGLR; QVREEFNTNLFVPGSK; EEFNTNLFVPGSK; DGLNVELDK; NESCGGHFREEHQTPEGEAAR; DDENYFYVGCWK; YQGNDTTAPELIKEPLEYEAIK
	NqrA	19	RGLDINLIGK; GLDINLIGK; GLDINLIGKAEEK; AEEKLTELSK; CEEFALVPEAFVGITPK; VVVHEGDVVK; AGDALFVNK; ADLNQQYVDFGIK; NVDELTSEEVK; NVDELTSEEVKK; DMPLAGNFEYELK; VYLGVGK; NQSNQTLLSAK; NAEVNVFDGK; CPAGNVGVQINHIDPVNK; DPSYVEVLVGTPLK; HMIMSGEYDK; VLPMDIYSEYLIK; EGLNTLR
	FrdB	14	NISFTIK; QNGPTAQGHFDTHEMK; GATTCQLYMR; RFNDGDVITVEPWR; FNDGDVITVEPWR; SAAFPVIK; SAAFPVIKDCMVDR; SAFDKIIAAGGYNTIR; IIAAGGYNTIR; TGQAQDANALLISK; NGSAMLFLSSK; VSQLALLPQGRPEAAK; MEELGFGNCTNTR; NESIANIAR
	RnfC	10	QAIFMLSQHIGAPAK; IDNVYDATGYR; VAGVTGMGGAGFPTFIK; GFIGIEENKPEAIR; LLTELCK; EHAGIEVVPLK; QLVDAVVGR; HIKNPSNFLVR; GTNAITVLTGK
	NqrF	7	VTITINGDQK; VSDAILNVK; NVSSFIK; NFNILSLK; VMMSGPYGDFHPNFTSGK; EMIWIGGGAGMAPLR; YYAGFAVNCVR
	NqrC	7	ALKPMQDANEALDK; KQILYSLNIR; QILYSLNIR; NLNNAETEETYAK; GTIYTCNIDGK; DGVTNEIALSVKK; GLGQYLDFLNQK
	RnfG	5	TLGAAVESVTGGFGGDLK; AGDWFQK; NPADGDLHVSK; NPADGDLHVSKDDK; AINQAYAVYIK

	NqrB	2	TSQSGVSIHDAIDSK; TSQSGVSIHDAIDSKR
	RnfD	2	LIVSLSPHAHGNDSVER; TGDASLLDR
**2**	NqrA	28	RGLDINLIGK; GLDINLIGK; GLDINLIGKAEEK; AEEKLTELSK; CEEFALVPEAFVGITPK; VVVHEGDVVK; AGDALFVNK; FASPVSGTVTAIVR; ADLNQQYVDFGIK; NVDELTSEEVK; VDELTSEEVKK; SLLDAGLFGYMNQLPYAIATNPSTTPK; AIFVSALR; DMPLAGNFEYELK; GNEEAFQTGISALSK; VYLGVGK; NQSNQTLLSAK; NAEVNVFDGK; CPAGNVGVQINHIDPVNK; KVAVAGSEVKDPSYVEVLVGTPLK; VAVAGSEVKDPSYVEVLVGTPLK; DPSYVEVLVGTPLK; ILNGNPLTGIK; AYNLDAR; HMIMSGEYDK; VLPMDIYSEYLIK; EGLNTLR; EGLNTLRK
	FrdA	18	IPEGPVAEK; AHSIAAQGGINAAK; NYQNDGDSVYR; LFYDTVK; LAEVSNNIIDQCVAQGVPFAR; EYGGMLANR; YEMEDVVIVDGR; RYPAFGNLVPR; LGLDEIMOR; VPTTAPEFDEAEK; VPTTAPEFDEAEKGVMAEIDR; TKEGLEEGLR; DGLNVELDK; NESCGGHFREEHQTPEGEAAR; YQGNDTTAPELIKEPLEYEAIK
	RnfC	14	IGGVHPEENKITAEIPTK; QAIFMLSQHIGAPAK; VGTLLAEAGGFVSAPVYSSVSGTVAK; SDTLETLAAHPELTSSEIVNR; VAGVTGMGGAGFPTFIK; VDKGFIGIEENKPEAIR; LLTELCK; EHAGIEVVPLK; QLVDAVVGR; HIKNPSNFLVR; VLAGGPMMGK; GTNAITVLTGK; GAVMGIIR
	NqrF	12	VTITINGDQK; CQVLEGGGEILDTEK; VSDAILNVK; VALPPGEHMDFIPGSYAQIK; IPAYDCIDYDK; IPAYDCIDYDKDFDK; ELIGEEYIGAWK; AYSMANYPAEGDIITLTVR; VMMSGPYGDFHPNFTSGK;EMIWIGGGAGMAPLR; EMHFFYGAR; YYAGFAVNCVR
	NqrC	11	ALKPMQDANEALDK; ALKPMQDANEALDKK; KQILYSLNIR; QILYSLNIR; NLNNAETEETYAK; GTIYTCNIDGK; TKYVFTVK; DGVTNEIALSVKK; DGVTNEIALSVK; DKGLGQYLDFLNQK; GLGQYLDFLNQK
	FrdB	10	NISFTIK; QNGPTAQGHFDTHEMK; GATTCQLYMR; SAAFPVIK; IIAAGGYNTIR; TGQAQDANALLISK; NGSAMLFLSSK; VSQLALLPQGRPEAAK; MEELGFGNCTNTR; NESIANIAR
	RnfG	6	TLGAAVESVTGGFGGDLK; AGDWFQK; NPADGDLHVSK; GNIIGKNPADGDLHVSK; NPADGDLHVSKDDK; AINQAYAVYIK
	NqrB	3	IKPNFEEGGK; TSQSGVSIHDAIDSK; TSQSGVSIHDAIDSKR
	RnfD	3	LIVSLSPHAHGNDSVER; TGDASLLDR; MSFGGLGNNPFNPALVGR
	NqrD	1	ITPDMVR
	NqrE	1	MEHAISLFFK

**3**	FrdA	26	SQIDSKIPEGPVAEK; RAHSIAAQGGINAAK; AHSIAAQGGINAAK; NYQNDGDSVYR; LFYDTVK; LAEVSNNIIDQCVAQGVPFAR; EYGGMLANR; SFGGAQVSR; YEMEDVVIVDGR; NLVSGKLER; LTLMSESLR; GEINPWDIAEEDR; RYPAFGNLVPR; YPAFGNLVPR; LGLDEIMOR; VPTTAPEFDEAEK; GVMAEIDR; TKEGLEEGLR; EGLEEGLR; EEFNTNLFVPGSK; DGLNVELDK; NESCGGHFREEHQTPEGEAAR; DDENYFYVGCWK; YQGNDTTAPELIKEPLEYEAIK; YQGNDTTAPELIK
	NqrA	23	RGLDINLIGK; GLDINLIGK; GLDINLIGKAEEK; AEEKLTELSK; VVVHEGDVVK; AGDALFVNK; FASPVSGTVTAIVR; ADLNQQYVDFGIK; NVDELTSEEVK; NVDELTSEEVKK; DMPLAGNFEYELK; GNEEAFQTGISALSK; VYLGVGK; NQSNQTLLSAK; NAEVNVFDGK; CPAGNVGVQINHIDPVNK; VAVAGSEVKDPSYVEVLVGTPLK; VAVAGSEVK; DPSYVEVLVGTPLK; AYNLDAR; HMIMSGEYDK; VLPMDIYSEYLIK; EGLNTLR
	FrdB	13	NISFTIK; QNGPTAQGHFDTHEMK; GATTCQLYMR; FNDGDVITVEPWR; SAAFPVIK; SAAFPVIKDCMVDR; SAFDKIIAAGGYNTIR; IIAAGGYNTIR; TGQAQDANALLISK; NGSAMLFLSSK; VSQLALLPQGRPEAAK; MEELGFGNCTNTR; NESIANIAR
	NqrF	9	VTITINGDQK; CQVLEGGGEILDTEK; VSDAILNVK; NVSSFIK; VMMSGPYGDFHPNFTSGK; EMIWIGGGAGMAPLR; EMHFFYGAR; YYAGFAVNCVR
	NqrC	9	ALKPMQDANEALDK; ALKPMQDANEALDKK; KQILYSLNIR; QILYSLNIR; NLNNAETEETYAK; GTIYTCNIDGK; TKYVFTVK; DGVTNEIALSVKK; DGVTNEIALSVK
	RnfC	7	IDNVYDATGYR; LLTELCK; EHAGIEVVPLK; QLVDAVVGR; NPSNFLVR; GTNAITVLTGK; GAVMGIIR
	RnfG	4	TLGAAVESVTGGFGGDLK; NPADGDLHVSK; NPADGDLHVSKDDK; AINQAYAVYIK
	NqrB	3	IKPNFEEGGK; TSQSGVSIHDAIDSK;TSQSGVSIHDAIDSKR
	RnfD	1	TGDASLLDR
**4**	NqrA	25	RGLDINLIGK; GLDINLIGK; GLDINLIGKAEEK; AEEKLTELSK; VVVHEGDVVK; AGDALFVNK; FASPVSGTVTAIVR; ADLNQQYVDFGIK; NVDELTSEEVK; NVDELTSEEVKK; AIFVSALR; DMPLAGNFEYELK; GNEEAFQTGISALSK; VYLGVGK; NQSNQTLLSAK; NAEVNVFDGK; CPAGNVGVQINHIDPVNK; VAVAGSEVKDPSYVEVLVGTPLK; VAVAGSEVK; DPSYVEVLVGTPLK; ILNGNPLTGIK; HMIMSGEYDK; VLPMDIYSEYLIK; EGLNTLR; EGLNTLRK
	FrdA	18	IPEGPVAEK; AHSIAAQGGINAAK; NYQNDGDSVYR; LFYDTVK; EYGGMLANR; SFGGAQVSR; YEMEDVVIVDGR; NLVSGKLER; LTLMSESLR;

			YPAFGNLVPR; LGLDEIMOR; VPTTAPEFDEAEK; GVMAEIDR; TKEGLEEGLR; EEFNTNLFVPGSK; DGLNVELDK; YQGNDTTAPELIK
	NqrF	12	VTITINGDQK; CQVLEGGGEILDTEK; VKNDLSIK; EWECTVISNK; NVSSFIK; IPAYDCIDYDK; NFNILSLK; AYSMANYPAEGDIITLTVR; VMMSGPYGDFHPNFTSGK; EMIWIGGGAGMAPLR; EMHFFYGAR; YYAGFAVNCVR
	NqrC	9	ALKPMQDANEALDK; ALKPMQDANEALDKK; KQILYSLNIR; QILYSLNIR; NLNNAETEETYAK; GTIYTCNIDGK; TKYVFTVK; DGVTNEIALSVKK; DGVTNEIALSVK
	FrdB	7	NISFTIK; IIAAGGYNTIR; TGQAQDANALLISK; NGSAMLFLSSK; VSQLALLPQGRPEAAK; MEELGFGNCTNTR; NESIANIAR
	RnfC	7	ITAEIPTK; IDNVYDATGYR; LLTELCK; EHAGIEVVPLK; NPSNFLVR; GTNAITVLTGK; GAVMGIIR
	RnfG	4	AGDWFQK; NPADGDLHVSK; NPADGDLHVSKDDK; AINQAYAVYIK
	NqrB	3	IKPNFEEGGK; TSQSGVSIHDAIDSK; TSQSGVSIHDAIDSKR
	RnfD	1	TGDASLLDR

Table S6: Mass spectrometric analyses of NQR, QFR and RNF subunits identified from *P. bryantii* membranes solubilized with 2.5 % DDM. Solubilisate was separated first on BN PAGE. The corresponding lane was further separated by 2D SDS PAGE. Spots exhibiting in gel fluorography were cut out and subjected to mass spectrometry (fig. 4B). Identified subunit, total number of identified peptides, and peptide sequences are presented.

**Box number**	**Identified protein**	**Peptide number**	**Peptide sequences**
**5**	FrdA	27	SQIDSKIPEGPVAEK; IPEGPVAEK; IPEGPVAEKWTNYK; RAHSIAAQGGINAAK; AHSIAAQGGINAAK; NYQNDGDSVYR; LFYDTVK; EYGGMLANR; SFGGAQVSR; YEMEDVVIVDGR; NLVSGKLER; LTLMSESLR; RYPAFGNLVPR; YPAFGNLVPR; LGLDEIMOR; VPTTAPEFDEAEK; GVMAEIDR; GVMAEIDRMNIK; TKEGLEEGLR; EGLEEGLR; QVREEFNTNLFVPGSK; EEFNTNLFVPGSK; DGLNVELDK; YQGNDTTAPELIKEPLEYEAIK
	FrdB	10	QNGPTAQGHFDTHEMK; GATTCQLYMR; FNDGDVITVEPWR; SAAFPVIK; SAAFPVIKDCMVDR; TGQAQDANALLISK; NGSAMLFLSSK; VSQLALLPQGRPEAAK; MEELGFGNCTNTR; NESIANIAR
	NqrA	5	AGDALFVNK; GNEEAFQTGISALSK; VYLGVGK; NQSNQTLLSAK; NAEVNVFDGK
	RnfC	5	ITAEIPTK; IDNVYDATGYR; QLVDAVVGR; NPSNFLVR; GTNAITVLTGK
	NqrF	4	CQVLEGGGEILDTEK; VSDAILNVK; EMIWIGGGAGMAPLR; YYAGFAVNCVR
	NqrC	1	NLNNAETEETYAK
	NqrB	1	TSQSGVSIHDAIDSK
**6**	FrdA	22	SQIDSKIPEGPVAEK; IPEGPVAEK; RAHSIAAQGGINAAK; AHSIAAQGGINAAK; NYQNDGDSVYR; LFYDTVK; LFYDTVKGGDYR; EYGGMLANR; SFGGAQVSR; YEMEDVVIVDGR; RYEMEDVVIVDGR; YGNLFEMYEEITDVFPGELGK; VPTTAPEFDEAEK; TKEGLEEGLR; EGLEEGLR; EEFNTNLFVPGSK; DGLNVELDK; NESCGGHFREEHQTPEGEAAR; YQGNDTTAPELIKEPLEYEAIK
	NqrA	22	RGLDINLIGK; GLDINLIGKAEEK; VVVHEGDVVK; AGDALFVNK; FASPVSGTVTAIVR; VRADLNQQYVDFGIK; ADLNQQYVDFGIK; NVDELTSEEVK; NVDELTSEEVKK; AIFVSALR; DMPLAGNFEYELK; VYLGVGK; VYLGVGKNQSNQTLLSAK; NQSNQTLLSAK; NAEVNVFDGK; VAVAGSEVKDPSYVEVLVGTPLK;

			VAVAGSEVK; AILEGNLKQTEHVR; ILNGNPLTGIK; AYNLDAR; HMIMSGEYDK; EGLNTLRKENA
	RnfC	17	IGGVHPEENKITAEIPTK; ITAEIPTK; ITAEIPTKVAELPK; QAIFMLSQHIGAPAK; VKVAGVTGMGGAGFTPFIK; VAGVTGMGGAGFTPFIK; VDKGFIGIEENKPEAIR; GFIGIEENKPEAIR; LLTELCK; EHAGIEVVPLK; QLVDAVVGR; HIKNPSNFLVR; AVISLDVPVCK; GTNAITVLTGK; GTNAITVLTGKDAHRK; GAVMGIIR
	NqrF	7	VTITINGDQK; VSDAILNVK; NVSSFIK; AYSMANYPAEGDIITLTVR; VMMSGPYGDFHPNFTSGK; EMIWIGGGAGMAPLR; YYAGFAVNCVR
	NqrC	6	KQILYSLNIR; QILYSLNIR; NLNNAETEETYAK; TKYVFTVK; DGVTNEIALSVKK
	FrdB	4	IIAAGGYNTIR; TGQAQDANALLISK; MEELGFGNCTNTR; LNREYISAK
	NqrB	3	IKPNFEEGGK; TSQSGVSIHDAIDSK; TSQSGVSIHDAIDSKR
**7**	FrdA	8	IPEGPVAEK; NYQNDGDSVYR; SFGGAQVSR; YEMEDVVIVDGR; LTLMSESLR; LGLDEIMQR; TKEGLEEGLR; EGLEEGLR
	FrdB	5	IIAAGGYNTIR; TGQAQDANALLISK; NGSAMLFLSSK; MEELGFGNCTNTR; NESIANIAR
	NqrA	3	VVVHEGDVVK; VYLGVGK; NQSNQTLLSAK
	NqrC	3	ALKPMQDANEALDK; NLNNAETEETYAK; GTIYTCNIDGK
	NqrB	2	SALKNYLNK; IKPNFEEGGK
	NqrF	1	VSDAILNVK
	RnfD	1	TGDASLLDR
**8**	FrdA	8	NYQNDGDSVYR; NLVSGKLER; LTLMSESLR; LGLDEIMQR; VPTTAPEFDEAEK; TKEGLEEGLR; DGLNVELDK
	NqrC	3	ALKPMQDANEALDK; NLNNAETEETYAK; GTIYTCNIDGK
	NqrA	3	AGDALFVNK; VYLGVGK; NQSNQTLLSAK
	FrdB	1	IIAAGGYNTIR
	RnfD	1	TGDASLLDR

References

1. Hein S, Klimmek O, Polly M, Kern M, Simon J. 2017. A class C radical S-adenosylmethionine methyltransferase synthesizes 8-methylmenaquinone. Mol Microbiol. 104:449–462. doi:10.1111/mmi.13638.
2. Chang KY, Yang J-R. 2013. Analysis and prediction of highly effective antiviral peptides based on random forests. PLoS ONE 8:e70166. doi:10.1371/journal.pone.0070166.
